# How Elephant Seals (*Mirounga leonina*) Adjust Their Fine Scale Horizontal Movement and Diving Behaviour in Relation to Prey Encounter Rate

**DOI:** 10.1371/journal.pone.0167226

**Published:** 2016-12-14

**Authors:** Yves Le Bras, Joffrey Jouma’a, Baptiste Picard, Christophe Guinet

**Affiliations:** Centre d'Etude Biologiques de Chizé, UMR, CNRS-ULR, France; University of Western Australia, AUSTRALIA

## Abstract

Understanding the diving behaviour of diving predators in relation to concomitant prey distribution could have major practical applications in conservation biology by allowing the assessment of how changes in fine scale prey distribution impact foraging efficiency and ultimately population dynamics. The southern elephant seal (*Mirounga leonina*, hereafter SES), the largest phocid, is a major predator of the southern ocean feeding on myctophids and cephalopods. Because of its large size it can carry bio-loggers with minimal disturbance. Moreover, it has great diving abilities and a wide foraging habitat. Thus, the SES is a well suited model species to study predator diving behaviour and the distribution of ecologically important prey species in the Southern Ocean. In this study, we examined how SESs adjust their diving behaviour and horizontal movements in response to fine scale prey encounter densities using high resolution accelerometers, magnetometers, pressure sensors and GPS loggers. When high prey encounter rates were encountered, animals responded by (1) diving and returning to the surface with steeper angles, reducing the duration of transit dive phases (thus improving dive efficiency), and (2) exhibiting more horizontally and vertically sinuous bottom phases. In these cases, the distance travelled horizontally at the surface was reduced. This behaviour is likely to counteract horizontal displacement from water currents, as they try to remain within favourable prey patches. The prey encounter rate at the bottom of dives decreased with increasing diving depth, suggesting a combined effect of decreased accessibility and prey density with increasing depth. Prey encounter rate also decreased when the bottom phases of dives were spread across larger vertical extents of the water column. This result suggests that the vertical aggregation of prey can regulate prey density, and as a consequence impact the foraging success of SESs. To our knowledge, this is one of only a handful of studies showing how the vertical distributions and structure of prey fields influence the prey encounter rates of a diving predator.

## Introduction

Foraging behaviour and more specifically, foraging success, is critical to the growth, reproduction and survival of animals and is therefore subject to natural selection [1]. As a result, foraging behaviour is expected to be optimized so that net energy gains are maximized for a given level of time and energy spent foraging [2–4]. While foraging at sea, diving predators are central place foragers from the ocean’s surface, where they need to come back to breathe between dives required to reach their prey at depth [5,6]. Under such constraints, the efficient diving behaviour of a predator is presumably the key to the optimization of their energy balance.

As the movements of free-ranging animals reflect how they interact with their physical and biological environment, spatial patterns in their trajectories provide a basis from which to understand foraging behaviour as well as gain insights on prey distribution and accessibility [6,7]. Recent technological advances in miniaturised electronic devices have enabled the detection of foraging events and the description of diving behaviour over very fine scales. Indeed, tri-axial jaw-mounted [8–10] or head-mounted [11,12] accelerometers has been successfully used to detect Prey Encounter Events (PEE hereafter) in several diving species of pinniped. Pitch angle, derived from three-dimensional acceleration data, provides spatial information on the vertical movements which cannot be obtained from time-depth dive profiles. As such, pitch angle can be helpful in addition to dive profiles for interpreting the diving behaviour of diving predators [13,14]. Three-dimensional magnetometry allows the computation of heading angle which, similarly to pitch angle according to vertical movement, complements time-depth data with information on horizontal movements. In this study, the spatial information obtained from pressure, accelerometer and magnetometer data are used to quantitatively assess how changes in vertical and horizontal diving behaviours relate to prey encounter rate.

Without acceleration data, accurate feeding indices are often difficult to obtain. A large number of studies use changes in surface GPS track patterns [15–18] or diving behaviour [19–22] as foraging indices. In various predator species, resource acquisition has been linked to a type of behaviour called area-restricted search (ARS) [23]. In a patchy environment, such as the open ocean, an animal will intensify its foraging in response to an increase in prey density [17]. Therefore, ARS is characterized by a decrease in displacement speed and an increase in track sinuosity in areas of putative prey aggregation [23,24].

Similarly, according to the vertical dimension, diving seabirds, marine mammals and leatherback turtles are expected to adjust their diving behaviour according to the quality and depth of the targeted prey patch [25–27]. A number of species, such as penguins and pinnipeds, perform behavioural adjustments in relation to prey density, such that they modulate the duration of the bottom phase of a dive [28–31]. The bottom phase of a dive has been validated as the time when most feeding occurs in several species including Antarctic fur seals (*Arctocephalus gazella*) [32,33], northern elephant seals (*Mirounga angustirostris*) [34], grey seals (*Halichoerus grypus*) [35], Magellanic penguins (*Spheniscus magellanicus*) [36], Weddell seals (*Leptonychotes weddellii*) [37] and leatherback turtles (*Dermochelys coriacea*) [38]. King penguins and macaroni penguins perform behavioural adjustments during the descent and ascent phases of their dives in relation to prey encounter rates during the previous dive [39,40], as predicted by Thompson and Fedak ([25]). Similarly, elephant seals increase their dive angles on putative foraging dives [14,41]. These adjustments take place mainly through changes in body angle, rather than through a change in swimming speed [40,42,43]. However, there is a lack of studies investigating quantitatively the mechanisms explaining how changes in diving behaviour (such as pitch angle adjustments during descent and ascent phases or pitch and heading variability during bottom phase) in response to prey encounter rate may impact the horizontal speed of the animal track at surface. Indeed, in most studies relating dive behaviour to horizontal speed measured from surface locations, no acceleration data were available and analyses were conducted on pressure-only dive metrics.

Living in the Southern Ocean, female Southern Elephants Seals (SES hereafter) with their large size and their great diving abilities are well suited for studying foraging behaviour, as using bio-logging tools causes minimal disturbance. SES spend 10 months a year at sea, covering thousands of kilometres during foraging trips and 90% of which is spend diving [44]. An average dive lasts between 20 and 30 minutes, at a depth ranging generally between 300–500 m, punctuated by surface periods of 2–3 minutes [45]. As for many diving predators, SES forage mostly at the bottom of their dives [6,11,46].

Female SES feed mainly on myctophids and cephalopods [47], both ecologically important groups of species within the Southern Ocean [48–51]. Because of its overall abundance and individual body mass, the SES is a major consumer of Southern Ocean marine resources [52,53]. As such, understanding better the foraging behaviour of data rich species such as SES [54] could provide valuable insight toward the biology and distribution of these important groups of species which are otherwise difficult to sample [55]. Prey abundance and density within the water column are likely to be key factors in the foraging success of these predators. As such, understanding the diving behaviour of SES in response to prey density could help to predict how changes in prey distribution may impact predator populations.

In this study, we examine (1) how diving behavior relates to prey encounter rates during the bottom phase of a dive, (2) the repercussions of these relationships on the travel transit rate of SESs and (3) their diving efficiency.

- To achieve (1), we used two metrics of diving behaviour describing (i) the vertical location of foraging activity through the water column (such as the depth of the bottom phase and its vertical extent through the water column) and (ii) fine-scale indicators of active foraging search (such as directional changes in pitch and heading angles). Then, we addressed a series of two sub-objectives. First, quantitatively measure the behavioural response of SES to changes in a proxy of prey density (prey encounter rate). Second, assess how this proxy relates to the vertical foraging location in the water column for a given level of active foraging search as it could help to predict how prey distribution may impact SES population in the future.
- Horizontal speed, measured using GPS locations at the surface, with the track sinuosity, is one of the main metrics used in state-space models to infer intensive foraging behaviour. Therefore objective (2) of the study is to understand, using fine scale information, how the vertical and horizontal diving behaviours related to prey encounter rate mechanistically translate into a change of horizontal speed.
- Our final objective (3) is to better understand the response of SES to the prey encounter rate in terms of foraging strategy, by investigating how adjustments in diving behaviour, and particularly diving angle, benefit diving efficiency, as indicated by the proportion of a dive’s total duration dedicated to the bottom phase.

## Materials and Methods

### Ethic statement

All fieldwork involving SES was approved and authorized by the ethics committee of the French Polar Institute (Institut Paul Emile Victor—IPEV) in May 2008. This Institute does not provide any permit number or approval ID, however animals were handled and cared for in total accordance with the guidelines and recommendations of this committee (dirpol@ipev.fr).

### Animal handling and electronic devices

During the breeding seasons (October and November) of 2010 through to 2014, a total of 9 female SESs of the Kerguelen Islands (49° 20’ S, 70° 20’ E) were equipped with (1) a Daily Diary tag (TDR10-DD, Wildlife Computers™, USA) and (2) a location collector device. The location detector device was either a Conductivity-Temperature-Depth satellite-relay data logger (CTD-SRDL, Sea Mammal Research Unit—University of St Andrew), a Time-Temperature-Depth Fastloc GPS data logger (SPLASH10-F, Wildlife Computers™, USA) or a Smart Position or Temperature Transmitting tag (SPOT, Wildlife Computers™, USA). Animals were captured with a canvas head-bag and anesthetized using a 1:1 combination of Tiletamine and Zolazepam (Zoletil 100) injected intravenously [56]. A TDR10-DD was then glued on each seal’s back and the location collector devices to the head using quick-setting Araldite (Araldite AW 2101). One individual was equipped with an additional accelerometer on its head. Details about the length and weight of each individual, logger-type deployment details are provided in the Table A in S1 Appendix.

The TDR10-DD logs depth (range = 0 to 2000 m, resolution = 0.5 m, accuracy = 1% of reading value, sampling frequency = 1Hz), temperature (range = -40°C to +60°C resolution = 0.05°C, accuracy = 0.1°C, sampling frequency = 1 Hz), and light (range = 5.10^−10^ W.cm^-2^ to 5.10^−2^ W.cm^-2^ (8 decades), resolution = 20 units per decade, accuracy = 0.1°C, sampling frequency = 1 Hz) as well as tri-axial acceleration (range = –2 g to +2 g, resolution = 0.05 m.s^-2^, sampling frequency = 16 Hz), tri-axial magnetometry (direction and strength of local magnetic field vector, range = -100 nTesla to +100 nTesla, resolution = 0.2 nTesla, sampling frequency = 16 Hz), and velocity (as the relative speed of the logger in surrounding water). The velocity sensor did not function correctly because of a build-up of dirt shortly after deployments which obstructed the propeller. Acceleration and magnetometry were measured along the same axes of the logger which were: (1) longitudinal (positive forward), (2) lateral (positive rightward) and (3) vertical (positive downward). TDR10-DD’s were positioned so that the logger’s X and Y axes approximately match longitudinal and lateral midlines of the SES.

The SPLASH10-F was the other type of data logger used in this study. They provided GPS locations 60% of the times the SES were back at surface to breathe. SPLASH10-F also measures depth, temperature and light (as described for the TDR10-DD) but these data were only used to synchronize with datasets from other loggers when it was necessary (when comparing head and back mounted accelerometers on a same individual, Figure A in Appendix S2). The CTD-SRDL and SPOT tags provided Argos locations (along with salinity and temperature in the case of CTD-SRDLs) that were used to locate the seals and retrieve the tags when they were back on land (but oceanographic data were not in this study).

### Acceleration and magnetometry data processing

All data processing was performed using R version 3.1.1 [57]. The majority of acceleration and magnetometry data analyses used in this study were done using the R package “rbl” (unless otherwise stated), available online at [58].

### Prey Encounter Events

The detection of Prey Encounter Events (PEE) was performed following Guinet *et al*. ([6]) and Vacquié-Garcia *et al*. ([59]). Dynamic accelerations, resulting from rapid head movements, were extracted from the longitudinal, lateral and vertical axes of the logger using an order 3 high-pass digital Butterworth filter with a normalized cut-off frequency of 2.64 Hz (performed with the signal package [60]). For each axis, a one-second fixed window was used to calculate the standard deviation. Signals were then processed using a moving standard deviation across a window of five seconds. Finally, a two-mean clustering was performed for each signal to distinguish “high state” from “low state”. A PEE occurred when the three axes were simultaneously in "high state" (see [59] for graphical illustration of the method). A continuous succession of "high state" was considered as a single PEE. One individual had both head-mounted and back-mounted accelerometers, so we used these data to check that these two acceleration data resulted in similar results (Pearson’s product moment correlation coefficient = 93%, see Figure B in S2 Appendix).

### Body posture angles

Pitch and roll describe the body posture of a SES with respect to the direction of the earth gravity vector whilst heading angle is in reference to the earth magnetic vector. Static acceleration is caused by the position of the gravity center of an animal compared with the gravity vector, which is always vertically orientated and can be used to infer pitch and roll angles. Static acceleration was obtained with an order 3 low-pass digital Butterworth filter with a normalized cut-off frequency of 0.20 Hz applied to the three axes as described in Richard *et al*. ([61]). The filtered output was then scaled to a unitary norm (function static_acceleration from the rbl package) so that pitch and roll angles could be computed directly from trigonometry formulas. Pitch and Roll angles were then calculated from this static acceleration, expressed in the North-East-Down (NED) frame of reference, using the pitch and roll functions from the animalTrack package [62]. The low-pass filter used to obtain static acceleration was applied to the magnetic data as well. Heading angle was then calculated (using the tilt_compensate function from the animalTrack package) from the pitch and roll angles alongside the filtered magnetic data expressed in the NED frame of reference.

### Swimming effort

The frequency spectrum of the lateral acceleration displayed a clear bimodality (see [63]). The high-frequency peak corresponds to the dynamic acceleration due to tail movements [64,65] which was extracted using an order 3 band-pass (from 0.44 Hz to 1.02 Hz) digital Butterworth filter [61,64–66]. To measure the frequency and magnitude of these tail movements, the absolute value of the resulting signal is then averaged to 1 Hz. We called the latter “swimming effort” and used it as a proxy of the cost of locomotion. This method is implemented in the swimming_effort function of the “rbl” package.

### Dive analyses

#### Dives

We defined dives as periods where animals were continuously deeper than 15 m under the surface. Because there is drift in the pressure readings of the tags over time, a zero offset correction of the depth time sequence was applied prior to the delimitation of dives (function offset_correction from the rbl package). SESs occasionally perform subsurface incursions, which results in a short number of atypical short and shallow dives. Moreover, unpredictable gaps in the time-depth sequence (due to a malfunction of the depth sensor) can sometimes cause different dives to be merged as a single very long one. According to the quantiles of all dive durations, dives lasting less than 8.33 min (500 s, Q1% = 511 s) or more than 32.50 min (1950 s, Q99% = 1947 s) were excluded in order to get rid of these irregular cases.

#### Dives phases

Each dive was divided into three phases: descent, bottom and ascent phases following Halsey *et al*. ([67]). This method defines the bottom of a dive as the period between the first and the last wiggle or step being deeper than a given depth threshold which is expressed as a percentage of the maximum depth in the dive. Steps and wiggles are time-depth patterns observed in the bottom of dives. Steps are defined as periods where the vertical velocity slows down but stays above 0 m/s while wiggles as periods where depth increases and then decreases, drawing a concave shape in the dive profile [67]. The upper limits of vertical velocity threshold applied to identify the steps in the time-depth dive profiles was kept to its value for king penguins (0.35 m/s, [67]) as it is close from observed values of non-swimming SES (see histogram of drift rates in [68]). In our datasets the large majority of PEE occurred deeper than 75% of the maximum dive depth (77.24% of all PEE) so this ledge threshold value from Halsey *et al*. ([67]) was kept. This method is implemented in the bottom_delim function of the “rbl” package. We choose this method to delimitate bottom phases of dives instead of the method we previously used in [28] to make sure that bottom phase limits could not fall within a step or a wiggle which would introduce mistakes when counting them. Indeed, by definition, the method developed by Halsey *et al*. ([67]) defines the limits of the bottom at the start and the end of such events.

### Dive statistics

A proxy of prey encounter density, PEE rate, was calculated as the total PEE of a bottom phase divided by its total duration in minutes. Our estimate of prey density is thus dependent on SES behavior. The diving behaviours to be used as explanatory variables were divided into two categories: variables related to the area of the water column targeted during the bottom phase and variables related to SES foraging activity, as described below.

#### Water column area targeted by elephant seals

The median depth at the bottom of dives is a standard variable to describe diving behaviour. During the bottom phase, a SES’s focus is expected to be on foraging whilst descent and ascent phases are primarily used for transit to this foraging ground. The median depth of the bottom phase reflects the vertical location of the resources on which the predator decided to forage on. As such, the bottom phase depth is positively related to the amount of time and energy that a SES spends to access their prey at depth. As the objectives of SESs are different in transit and in bottom phase, we focused on the bottom and selected the PEE rate at bottom as an index of the prey encounter density which is independent from the duration of the transit phases. In this study the bottom median depth is used to test whether or not the prey encounter density varies according to depth.

To describe in more detail the vertical location of the bottom phase, “bottom vertical extent” was defined as the depth range between the 10% and 90% depth quantile. Using the quantiles rather than extremes of depth yields a more robust measure of the vertical extent of the water column layer targeted by SES, excluding extreme values from unrealistic bottom phase delineation or atypical diving behaviour (where SES perform a high amplitude wiggle thus exploring a wide depth range but only for very short time).

#### Foraging activity

The number of wiggles has been used as a proxy of the foraging success for various diving predators (e.g. Northern Elephant Seals and King penguins [20,69]) and are also encountered in the bottom of SES dives. While wiggles are correlated to the number of PEE, the steps rather resemble a gliding pattern (slow ascending or descending vertical speed) and it is not clear yet if this diving pattern is associated with foraging or not. Percentage of the bottom duration during which SES were performing steps and wiggles were included in the analyses as they stand for two distinct diving behaviors describing the foraging activity during the bottom phase.

The mean descent and ascent pitch angle (circular mean, CircStats package [70]), as well as the average descent and ascent swimming effort were computed to account for the transit time adjustments made by the SES in response to the foraging success and to the targeted bottom depth. In a way to assess the amount of directional changes performed by SESs during the bottom phase of their dives, the circular variance ([69]) of the pitch and heading angles were calculated. The circular variance of the pitch and heading angles provide comparable indices of the sinuosity according to of the vertical (pitch angle) and horizontal (heading angle) dimensions. While pitch variance could be considered redundant with the percentage of time doing wiggles at bottom, it is actually complementary. Indeed for a given quantity of wiggles the greater the pitch variance the steeper they are. Another advantage of this measure over the wiggles is that it is a simple summary statistic of a quantitative variable and does not depend on an algorithm to detect specific events. Hence, it varies continuously, which results in a subtle description of the diving behaviour, and has no detection error issue.

Because our focus is on dives associated with foraging, drift dives, during which SES are resting and/or digesting [71], were removed from the dataset prior to the statistical analysis.

### Statistical analysis

We implemented five models (numbered 1a, 1b, 2, 3 and 4) relating to the objectives articulated in the introduction (numbered 1, 2 and 3).

The first objective is to describe the relationships between the PEE rate at the bottom (the response variable) and the diving behaviours during daytime (model 1a) and night-time (model 1b). This allows testing whenever the PEE rate varies according to depth independently of the effect of daily vertical migrations of the SES prey. Namely, the diving behaviours used as explanatory variables in these models are: the median depth at the bottom of dives, the bottom phase vertical extent, the proportion of bottom time spent doing wiggles or steps during the bottom phase, the mean pitch angle in the descent and ascent phases and the variances of pitch and heading angles. We used the number of PEE at the bottom as the response variable of a count model (log link) but actually modelled PEE rate as response by providing the log-transformed bottom duration as an offset variable (the effect of an offset variable is not estimated but forced to one). Poisson family GLMs indicated over-dispersion. We used the Negative Binomial family GLM (MASS package [72]) to address over-dispersion. The inter-individual differences in PEE rate were modelled by specifying the SES identities as fixed effects intercepts.

The second objective is to examine how the horizontal speed at surface during dives–measured from the distance and duration between SES locations taken as they surface before and after dives—relates to the diving behaviours (model 2). To have a reliable estimate of this surface horizontal speed, we used only dives where observed GPS locations preceding and following the dive (39% of dives). This subset introduces a bias toward the selection of dives with longer recovery time that we could not account for (surface periods lasting 127 s for located surfaces but 120 s otherwise, see Table A and Figure B in S3 Appendix for details). We used a Linear Mixed Model (LMM, nlme package [73]) with the horizontal speed at surface as the response variable, and the same diving behaviours used in models 1a and 1b as explanatory variables and an individual as a random intercept.

The third objective is to investigate the effects of diving behaviours on dive efficiency (model 3). Diving efficiency is defined as the bottom phase duration divided by the full dive duration. The proportions typically display more variability around their mean so we used a variable dispersion beta regression model (betareg package [74]) to handle the heteroskedastic nature of this response variable. The diving behaviours used as explanatory variables are the same as in previous models (models 1a, 1b and 2) and the SES identities were specified as fixed effects intercepts.

The last model implemented in this study (model 4) is related to the third objective and aims at clarifying how the transit between surface and the bottom phase location may be regulated by diving behaviour adjustment. As for model 2, we implemented a LMM. In this model, the response variable is the duration of the phase, and the explanatory variables are the type of phase (ascent or descent), the maximum depth reached during the phase and the average swimming effort during the phase. A random intercepts of SES individual was included. A single model was used for both types of transit phase (ascent or descent). We tested for interactions between the type of phase and other explanatory variables to enable the estimation of distinct relationships during ascent and descent phases.

The model selection procedure was performed in two stages. The first stage consisted in a stepwise AIC starting from the full model with all explanatory variables and dropping variables step by step until the AIC reached a minimum. In order to allow potentially non-linear relationships in the five models that we implemented, linear combinations of the powers of covariates (polynomials) were tested. These polynomials allow fitting a relationship of any shape but the more complex is the shape the more parameters it requires and the stronger it is penalized by AIC. Thus, the second stage of the model selection procedure was to test for non-linear relationships by computing the AIC with polynomials of the previously selected explanatory variables of increasing degree until the model AIC reached a new minimum. During the model selection, models were fitted with Maximum-Likelihood algorithm. Final models were re-fitted with Restricted Maximum Likelihood algorithm. For each of the five models the final set of explanatory variables selected is displayed on the corresponding figure. The specification of correlation structures (such as AR, ARMA or ARIMA) induces very large computation time and is not implemented for all types of model that we used. We addressed the temporal autocorrelation issue by selecting one dive every ten. The existence of an autocorrelation structure in the models’ residuals was assessed by plotting their auto-covariance function for each individual (performed with the acf function). Colinearity issues between covariates was checked prior to model selection using Variance Inflation Factor (VIF < 5, usdm package [75]).

Various pseudo-R^2^ were used to assess the amount of variation explained by the top models. For LMMs (models 2 and 4) we used an equivalent of the Ordinary Least-Squares (OLS) R^2^ which has been developed by Nakagawa and Schielzeth ([76], implemented in the MuMIn R package [77]). It has a “marginal” (R^2^m) and a “conditional” (R^2^c) component which can be interpreted as the variance explained by fixed effects only (R^2^m) and by the entire model (R^2^c). For GLMs (models 1a and 1b) we calculated the percentage of the null model deviance explained (D^2^, Table 1) by the top models as a substitute to OLS R^2^ [78]. Moreover, for these models, we calculated the null model deviance explained (hereafter abbreviated NDE) by each explanatory variable. Finally, to evaluate goodness-of-fit of the beta regression (model 3) and to compare it to the other models, we calculated a pseudo R^2^ metric defined as the squared Pearson’s correlation coefficient between observed values (transformed with the link function) and fitted values of linear predictor. This pseudo R^2^ metric (noted Pearson^2^ in Table 1) ranges from 0 to 1 and provides an indication of correlation between predicted values and actual values (where the closer to 1 the better).

**Table 1:** Goodness-of-fit of the top models as indicated by pseudo-R^2^. See the “Statistical analysis” section for details about these metrics.

Model	D^2^	Nakagawa *et al*. R^2^	Pearson^2^
1a (Neg. Bin. GLM)	58%		56%
1b (Neg. Bin. GLM)	63%		60%
2 (LMM)		R^2^m = 41%, R^2^c = 48%	47%
3 (Beta regression)			87%
4 (LMM)		R^2^m = 93%, R^2^c = 94%	85%

## Results

### Overall diving behaviour

A total of 20189 dives were recorded from the 9 post-breeding female SESs. Of these 8.4% were classified as drift dives. Mean dive duration was 18.38 min (1103 s) ± SD 5.13 min (308 s) and mean bottom duration was 7.95 min (477 s) ± SD 4.12 min (247 s). The overall average of mean depth at bottom was 409 m ± SD 192 m with a maximum of 1307 m. PEEs were detected in 91.1% of non-drift dives. 10.48% of PEE occurred in descents, 78.03% in bottom phases and 11.49% in ascent. Additional descriptive statistics of diving behaviours are available in the Table C in S1 Appendix.

### Relationships between prey encounter rate and diving behaviours

**Fig 1:**
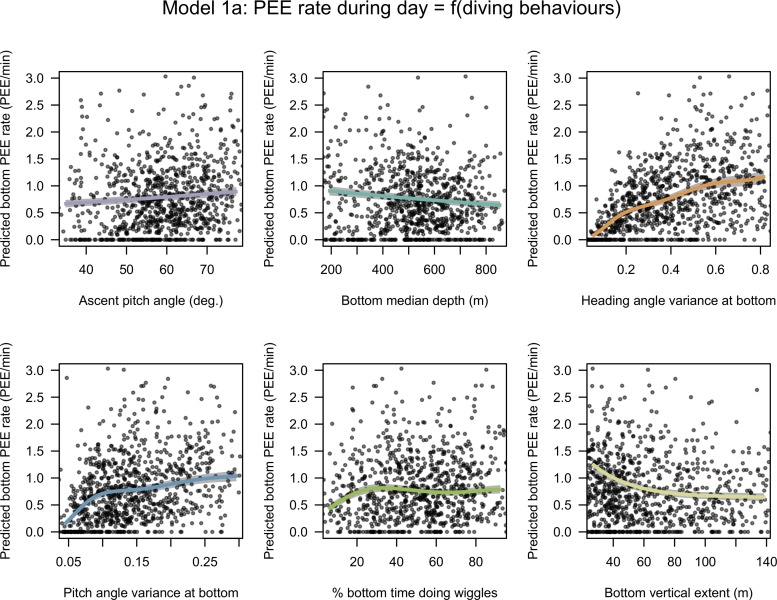
Estimated effects of covariates selected in model 1a. Expected response predicted with a covariate varying from the 5% to the 95% quantile of its observed values with other covariates at their mean. The x axes have been scaled to range from -2 to 2 normalized so that regression slopes are comparable but are annotated with raw units so that units are comprehensible. The grey shades around the regression lines indicate the standard error of the mean prediction estimates.

**Fig 2:**
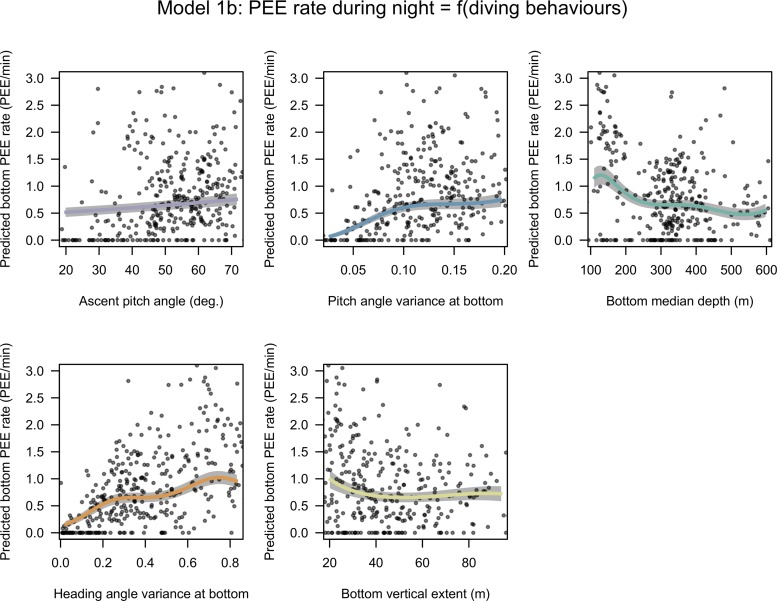
Estimated effects of covariates selected in model 1b. The predictions for each covariate varying from the 5% to the 95% quantile of observed values with other covariates at their mean. The x axes have been scaled to range from -2 to 2 normalized so that regression slopes are comparable but are annotated with raw units so that units are comprehensible. The grey shades around the regression lines indicate the standard error of the mean prediction estimates.

**Fig 3:**
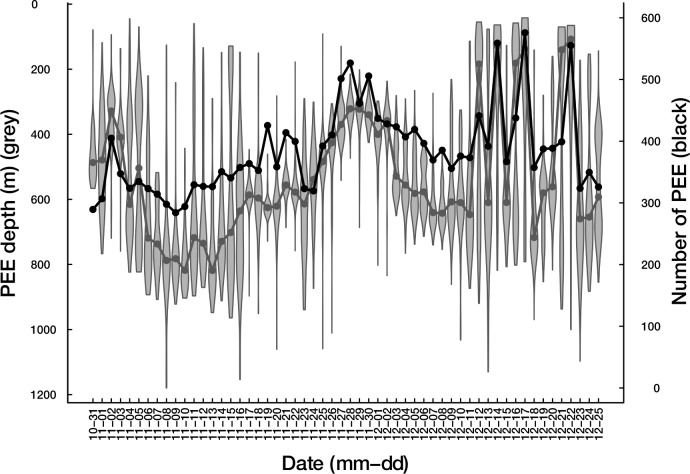
Time series of the vertical distribution of PEE in the bottom of dives and of the number of PEE rate (individual 2011–28). The black lines stand for the PEE rate (number of bottom PEE per day, daytime only), the grey violins for the vertical distribution of daytime bottom PEE (with median depth of daily PEE indicated by the grey dots).

The models 1a and 1b selected by the model selection process included ascent pitch diving angle, angular variances of pitch and heading during bottom phase, the median depth and vertical extent of bottom phase and, for model 1a only, the percentage of bottom time spent doing wiggle. The PEE rate at the bottom during day and night is positively related to ascent pitch diving angle (3% NDE for daytime and 11% NDE at night) and to the angular variances of pitch (5% NDE for daytime and 20% NDE at night) and heading (31% NDE for daytime and 6% NDE at night) angles at bottom (Figs 1 & 2). Negative relationships were found between PEE rate and the bottom median depth (10% NDE for daytime and 15% NDE at night) and to the bottom vertical extent (3% NDE for daytime and 1% NDE at night) (Figs 1–3). PEE rate at bottom responded to bottom time doing wiggles during day only (1% NDE).

According to the regression slopes, the strongest effects on prey encounter rate during the day are heading and pitch angle variances at the bottom (particularly for low values), the bottom vertical extent (again for low values) and the bottom median depth (Fig 1). Below 20% of bottom phase duration, the time at bottom spent doing wiggles has a large and positive effect on the PEE rate at bottom, but beyond this point the effect is weak (Fig 1). Similarly, the negative effect of the vertical extent of the bottom phase decreases from 60 m toward higher values (Fig 1).

Regression slopes for the night model (model 1b, Fig 2) display similar patterns to those of the day model. As in model 1a, the negative effect of the bottom vertical extent on the PEE rate at bottom decreases from low to high values going from a strong negative below 20 m, to an approximately flat relationship beyond this threshold (Fig 2). The regression line estimated for the bottom median depth differs from model 1a by displaying a non-linear shape and a steeper negative slope overall (Fig 2).

### Relationships between horizontal speed at surface and diving behaviours

**Fig 4:**
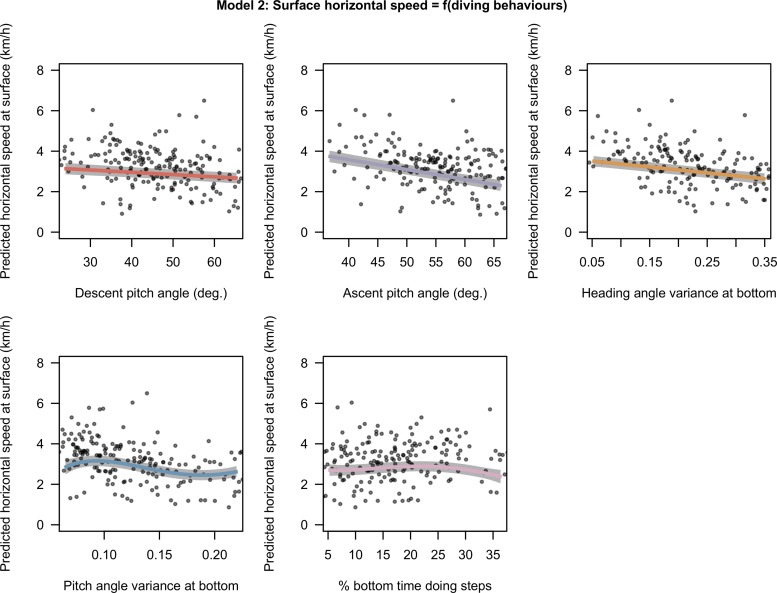
Estimated effects of covariates selected in model 2. The predictions for each covariate varying from the 5% to the 95% quantile of observed values with other covariates at their mean. The x axes have been scaled to range from -2 to 2 normalized so that regression slopes are comparable but are annotated with raw units so that units are comprehensible. The grey shades around the regression lines indicate the standard error of the mean prediction estimates.

The model 2 with average pitch diving angle in descent and ascent phases, angular variance of pitch and heading, and percentage of time spent doing steps was selected by the model selection process. Descent and ascent diving angles and variances of heading angle at bottom have a linear negative effect on the horizontal speed measured at surface with GPS locations (Fig 4). We found a negatively orientated non-linear effect of the pitch angle variance at bottom on the surface speed (Fig 4). Finally, the amount of time doing steps in the bottom has a low positive effect in the range of low values (< 25%, Fig 4) but a negative effect beyond 25% (Fig 4). The strongest slopes are observable for ascent pitch angle and heading variance at bottom (Fig 4).

### Relationships between diving efficiency and diving behaviours

**Fig 5:**
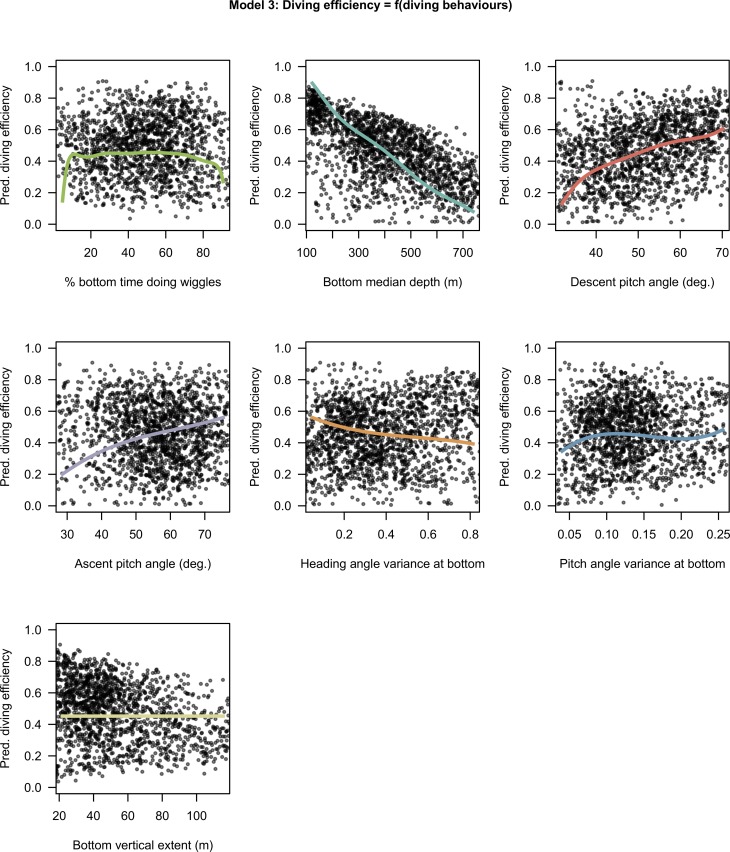
Estimated effects of covariates selected in model 3. The predictions for each covariate varying from the 5% to the 95% quantile of observed values with other covariates at their mean. The x axes have been scaled to range from -2 to 2 normalized so that regression slopes are comparable but are annotated with raw units so that units are comprehensible.

The model 3 with average pitch diving angle in descent and ascent phases, angular variance of pitch and heading, percentage of time spent doing wiggles and median depth in bottom phase was selected by the model selection process. The diving efficiency (proportion of dive time spent in the bottom phase) is positively related to descent and ascent pitch angle, and negatively related to the bottom median depth and the variance of heading angle at the bottom (Fig 5). In comparison to these diving behaviours, the bottom time spent doing wiggles, the bottom vertical extent and the variance of pitch angle at the bottom have weak effects (Fig 5).

**Fig 6:**
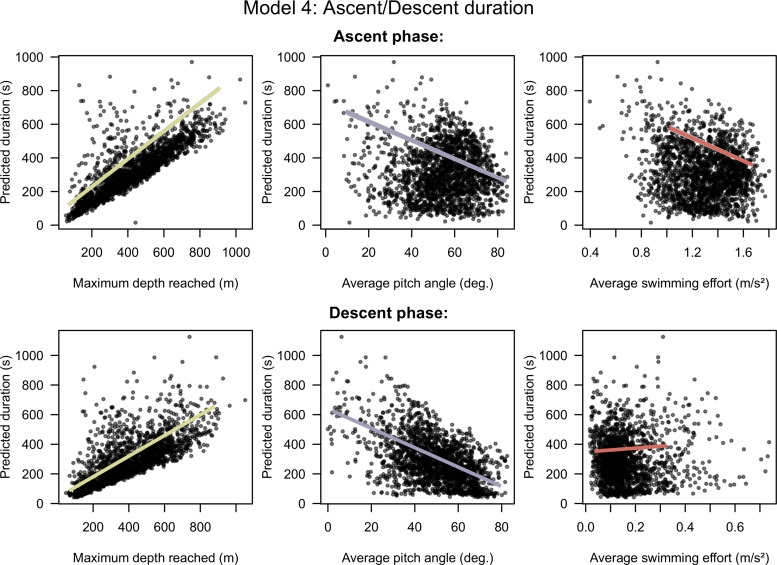
Estimated effects of covariates selected in model 4. The predictions for each covariate varying from the 1% to the 99% quantile of observed values with other covariates at their mean. The x axes have been scaled to range from -3 to 3 normalized so that regression slopes are comparable but are annotated with raw units so that units are comprehensible. The grey shades around the regression lines indicate the standard error of the mean prediction estimates.

The model 4 with average pitch diving angle, average swimming effort and maximum depth of the phases was selected by the model selection process. Descent and ascent durations are both negatively related to the steepness of pitch angles and positively related to the depth (Fig 6). The swimming effort is another significant driver of the ascent duration but not of the descent duration (Fig 6). While increasing the ascent vertical speed implies greater swimming effort per unit of time it is associated to smaller duration and consequently to a smaller amount of swimming effort cumulated over the complete ascent phase (Fig C & Table E in S4 Appendix).

### Models results

The datasets contained 950 complete observations for model 1a (30.64 observations per parameter) and 364 for model 1b (12.55 observations per parameter), 212 for model 2 (17.67 observations per parameter) and 1,823 for model 3 (41.43 observations per parameter). The pseudo-R^2^ used to assess the response variation explained by the top models (Nakagawa and Schielzeth’s R^2^ for LMMs, D^2^ for GLMs) or their goodness-of-fit (Pearson^2^ for beta regression) are provided in Table 1.

## Discussion

These results reveal that the prey encounter rate of SESs is driven by two main factors: (1) the depth (i.e. the vertical accessibility) of a prey patch from the surface, where the closer to the surface the better, and (2) the bottom vertical extent, where lower values were associated with higher PEE rate. This suggests that the prey catch rate of female SESs increased when well defined, narrow layers of high density prey were encountered. As such, the fine scale density within the water column, possibly rather than the overall prey density over the whole water column visited by the SES, appears to be one of the main drivers of SES prey catch rate. When high prey encounter rate is met, elephant seals adjust their diving behaviour by increasing both their descent and ascent angle (Figs 1 and 2), likely to minimize their transit time (Figs 5 and 6), and increase both their horizontal and vertical sinuosity during the bottom phase of their dive zigzagging within the prey patch layer (Figs 1 and 2). The negative relationship between the horizontal transit rate and putative feeding activity has been observed for numerous marine predators (SES [19] see also Figure A in S3 Appendix, northern elephant seals (*Mirounga angustirostris*) [79], wandering albatross (*Diomedea exulan*), antarctic fur seals (*Arctocephalus gazella*) [80]). A number of these variables that impacted on the PEE rate at bottom (models 1a and 1b) affected oppositely the horizontal speed measured at surface with GPS location (model 2). Namely, these variables were the diving angle in ascent phase and the variances pitch and heading angle during bottom phases (Figs 1, 2 and 4). As a consequence, the horizontal speed measured at surface decreases when encountering more prey.

### Behavioural adjustments to prey encounter rate

To reduce transit time and increase diving efficiency, SESs increase their vertical transit speed with steeper pitch angles mainly but also, in ascent, by increasing their swimming effort (Fig 6). We found that swimming effort had a greater importance on vertical speed in the ascent phase compared to the descent phase of the dives (Fig 6). This lower contribution of the swimming effort to the vertical speed during the descent is likely to be related to the negative buoyancy of post-breeding SES females, which tend to glide down to the bottom of their dive [61,66]. Indeed, the SES leaving Kerguelen after breeding are in poor condition and the post-breeding foraging trips do not last long enough to fully restore their lipid provisions. During the ascent phase, negatively buoyant female SES both increase their ascent angle and their swimming effort, with both factors having an equivalent contribution to explain the increased vertical transit speed (Fig 6). However, the overall swimming effort in response to an increased vertical speed is negative, with a greater swimming effort per unit of time being compensated by shorter transit duration (Figure C in S4 Appendix).

Steeper pitch angles in descent and ascent phases were found to slow down significantly the horizontal component of SES movement (model 2, Fig 4) and to increase the proportion of time the SES spent in bottom phase during their dives (model 3, Fig 5). Adjustments in diving angles lead to a trade-off between the amount of time the animal can spend to forage at depth and the horizontal speed. In an environment with a high prey density, steep diving angles allow individuals to spend longer time at depth in contact with prey and to remain in the same area for their next dives. However, in low prey density area, flat diving angles speed up horizontal transit rate and shorten search time to find a new prey patch. The positive relationship between the steepness of ascent angle and the PEE rate at bottom (model 1, Figs 1 & 2) could be interpreted according to the optimal foraging theory as a behavioural adjustment to maximize the time spent in high prey density environment. This relationship was weak in model 1a, suggesting that the deeper dives performed during daytime leave less room for such adjustments. As the diving angle in descent could be a way to regulate the horizontal speed it is likely to respond not only to the prey patch quality but also to the migration stage of the SES [43]. Oblique descent angles have also been suggested to relate to prey location where it could help the SES to combat the camouflage of squids in the downward light [41].

Diving depth had the strongest impact on the diving efficiency, which can be explained by the greater transit duration to reach those greater depths which reduces the amount of time SES could allocate to foraging at the bottom (model 3). The energetic cost of transit to the bottoms of dives is also related to the body buoyancy. Swimming energy expenditure is the lowest at neutral buoyancy allowing the seals to increase their diving efficiency [61]. Seals also tend to adjust their diving efficiency according to the foraging success of the current and the previous dives [28].

The prey encounter rate was positively related to the circular variances of both pitch and heading angles (Figs 1 and 2) which are indicative of the vertical and horizontal sinuosity of the bottom. These diving behaviours also impacted strongly on the horizontal speed at surface, thus increasing the residence time of SES in a given area. This is consistent with observations of Area Restricted Search behaviour in other diving seabirds and pinnipeds or, at larger spatial and temporal scales, with SES [19,68,81,82] alongside side optimal foraging theory which predicts longer residence in high prey density grounds. The heading angle variances at the bottom was detrimental to diving efficiency (model 3, Fig 5), suggesting that increased horizontal sinuosity is associated with prey chasing or handling and to a greater energy expenditure.

The bottom time spent performing wiggles was related to PEE rate at the bottom in day conditions. Its effect displayed a plateau at intermediate-high values (> 30% Fig 1). Wiggles were also found to impact on diving efficiency toward extreme values (Fig 5). Pitch and heading angular variances during the bottom phase of the dive had greater and more consistent effect over the complete range of observed values and appear to be a more reliable indicator of PEE rate. The bottom time spent doing steps related solely to horizontal speed at the surface and displayed contrasting effects (Fig 4), suggesting that this behaviour is employed for multiple purposes. From our perspective, steps may represent short drifting periods or gliding periods allowing horizontal travel at low expense or to locate prey during the bottom phase of the dive.

Della Penna *et al*. ([83]) showed that when foraging dynamic oceanographic mesoscale structures, such as eddies, the horizontal displacements of SES encountering a high PEE rate could be as passive as those of lagrangian drifters. Thus, the SES would keep in contact with the foraging resources by reducing their horizontal displacements. As currents are supposed to contribute significantly to the horizontal movements of SES when they forage intensively [83], the varying current strength encountered by the animal along its trajectory is a source of noise when trying to compute the actual horizontal speed of SES from satellite locations. Despite this methodological limitation, our results highlight that foraging intensive behaviours—increasing of descent and ascent diving angles, but also of horizontal and vertical sinuosity during the bottom phase of their dive—have a negative effect on the horizontal component of their movement. As a consequence they remain within the prey patch, but become more sensitive to current transportation [83,84] and are passively transported with the prey patch by the current. Under such a situation, with a high current velocity the ARS/non ARS behaviour detected from the surface track could result primarily from the current velocity field rather than from the active horizontal movements from the animals. As such, inference of foraging state of the animal using state space models could be erroneous.

### Area of the water column targeted by SES

SESs were more successful when foraging closer to the surface (model 1a and 1b). Either in day (Figs 1 & 3) or in night conditions (Fig 2), the greater the bottom depth the lower PEE per unit of time they were, showing that this observation cannot be explained by diel vertical migrations of SES prey. This negative relationship between the bottom depth and the prey encounter rate at the bottom could indicate a decrease in prey density, a change in prey type/size or a decrease of the ability of SES to catch prey items with increasing depth. Other studies have led to similar results. For example, [85] observed that the occurrence of bioluminescent events detected from light sensors carried by foraging elephant seals was negatively related to their diving depth. Williams & Koslow ([86]) sampled micronekton between the surface and a depth of 900 m with a mid-water trawl and found a decreasing micronekton biomass with depth at night and, in autumn, during daytime as well.

Predators should match their foraging effort to prey distribution. Seventy seven percent of all PEE took place in the bottom phase of a dive. With regards to the accessibility and abundance of resources, the bottom phase is valuable enough to motivate the SES to stop the descent phase and focus on the search of prey at depth. Once the bottom depth is reached, the vertical extent of the bottom phase is expected to provide an indication on the vertical distribution of prey. The PEE rate at the bottom was negatively related to the vertical span of the water column explored during the bottom phase (model 1, Figs 1–3), suggesting that the dispersion of prey along the vertical dimension regulates the prey density encountered at depth by SES. To our knowledge this is the first time that this relationship is highlighted and could be a novel aspect that is worth considering when investigating fine scale prey density underwater.

A range of diving behaviours (e.g. dive depth, descent speed an dive duration) have been associated with mesoscale oceanographic features such as cyclonic eddies, where SES exhibit shallower diving depth compared to anti-cyclonic eddies and other oceanographic domains [82,87]. Water temperature was found to have a direct influence on the diving depth of SES, with SES diving deeper in warmer waters to access their prey [6,82]. Furthermore, bottom depth was shown to be strongly negatively related to light intensity at depth which is attenuated by phytoplankton concentration within the euphotic layers [88]. Characteristics such as a high chlorophyll concentration and cold water at surface are observed in cold-core eddies [89,90], shown to be successful foraging areas where the SES dive depth is close from surface [87]. While underlying determinants leading SES to explore a narrow vertical depth range in their dive bottom phases remain unclear, one could hypothesize that similar bio-physical oceanographic processes could locally constrain prey to aggregate vertically in thinner yet denser prey layers. Future work could try to assess the validity of this hypothesis.

This study could only focus on the vertical dimension of prey distribution because, due to sensor malfunction, we did not have access to the actual swimming velocity when the seal was foraging at the bottom of its dive to allow for a more accurate description of the 3D spatial structure of the prey field. Without that information we could not estimate the volume of water prospected by SES and compare it to the number of PEE in order to get an indication of prey density independent from SES behaviour (in PEE/m^3^ instead of PEE per unit of SES bottom time). Additional studies may use 3D dive reconstructions [13,80,91–93] to determine if the effect of the vertical extent of the bottom on the foraging success would be related to the presence/absence of small scale schooling-prey patches (leading to a small vertical extent of the bottom) or conversely, due to changes at a larger scale in the vertical aggregation of prey layers within the water column. We believe that the findings of this study are likely to be generalized to other air breathing divers foraging on small prey items such as mesopelagic fishes or crustaceans, but differences are likely to be found for diving predators foraging on large prey items. Instead, the latter may abort their dive after catching very large preys to return to the surface to feed as observed in Weddell seals (*Leptonychotes weddellii*) feeding on Antarctic toothfish (*Dissostichus mawsoni*) [94].

The prey encounter events detected from acceleration data only provide quantitative information about prey. How the prey species and size relate to the diving behaviour and the decreasing prey encounter rate that we observed along depth remain unknown. Moreover, bio-logging data are collected by free-ranging diving predators which are unlikely to cover the entire range of habitat available to their prey. This makes it difficult to assess the deeper limit of the prey patch on which the SES feed at the bottom of their dives or to test predictions about their foraging behaviour. The development of video camera [34,91,95] and miniaturized sonar loggers [96] may help to overcome these difficulties by providing information about the prey quality and extend our perception range of environment surrounding the SES.

## Supporting Information

S1 Appendix Diving behaviour and device deployment details for the 9 post-breeding female SES. (PDF)

S2 Appendix Detection of Prey Encounter Events (PEE). (PDF)

S3 Appendix Surface horizontal speed. (PDF)

S4 Appendix Influence of vertical speed on swimming effort during transit phases. (PDF)
