# Supplementary material for: How Elephant Seals (Mirounga leonina) Adjust Their Fine Scale Horizontal Movement and Diving Behaviour in Relation to Prey Encounter Rate
Source: PLoS One. 2016 Dec 14;11(12):e0167226. doi: 10.1371/journal.pone.0167226 (PMC5156345; doi:10.1371/journal.pone.0167226)
Supplement: S1 Appendix — (PDF) [file pone.0167226.s001.pdf]

S1 Appendix: Diving behaviour and device deployment details for the 9 post-breeding female SES

I - Device deployment details

Table A: Device deployment

Individual	"TDR" device	Head/Back mounted	Location device *(all head-mounted)*	Weight *(kg)*	Length *(cm)*	Recording duration *(days)*
	TDR10-DD	Back	SPLASH10-F	425	278	71
2010-21	SPLASH10-F	Head				
	TDR10-ACC	Head				
2012-09	TDR10-DD	Back	SPLASH10-F	328	257	33
2012-14	TDR10-DD	Back	CTD-SRDL	258	236	19
2012-15	TDR10-DD	Back	CTD-SRDL	275	234	15
2013-10	TDR10-DD	Back	CTD-SRDL	300	244	50
2013-11	TDR10-DD	Back	CTD-SRDL	277	238	62
2013-16	TDR10-DD	Back	SPOT	236	227	17
2014-24	TDR10-DD	Back	CTD-SRDL	310	255	26
2014-35	TDR10-DD	Back	CTD-SRDL	200	216	23

Links to tags' manufacturer websites

Wildlife Computers tags:

TDR10-DD http://wildlifecomputers.com/our-tags/daily-diary/

SPLASH10-F http://wildlifecomputers.com/our-tags/splash/

SPOT http://wildlifecomputers.com/our-tags/spot/

TDR10-ACC (not commercialized anymore)

Sea Mammal Research Unit, University of St Andrews:

CTD-SRDL http://www.smru.st-andrews.ac.uk/Instrumentation/SRDL/

II - Dive statistics for all individuals and all dives together

n dives = 20189, 8.04% of drift dives.

Overall maxmimum depth recorded = 1307 m

Table B: Number of dive according to day or night

Number of dives in Day/Night
Day :10528
Night : 4176
Transition: 5320
NA's : 165

Table C: Descriptive statistics of diving behaviour variables

	Number of PEE at bottom	Bottom duration (s)
**nbr.val**	20189	20189
**nbr.null**	3844	0
**nbr.na**	0	0
**min**	0	1
**max**	42	4217
**range**	42	4216
**sum**	128391	9623049
**median**	5	462
**mean**	6.359	476.6
**SE.mean**	0.04451	1.74
**CI.mean.0.95**	0.08724	3.412
**var**	39.99	61159
**std.dev**	6.324	247.3
**coef.var**	0.9944	0.5188

	Horizontal speed at surface (km/h)	Median depth at bottom (m)
**nbr.val**	20015	20189
**nbr.null**	13	0
**nbr.na**	174	0
**min**	0	31.47
**max**	664.1	1203
**range**	664.1	1171
**sum**	102851	8251604
**median**	3.248	391.4
**mean**	5.139	408.7
**SE.mean**	0.0615	1.354
**CI.mean.0.95**	0.1205	2.654
**var**	75.71	37014
**std.dev**	8.701	192.4
**coef.var**	1.693	0.4707

	Bottom vertical extent (m)	Bottom time doing wiggles (%)
**nbr.val**	20189	20189
**nbr.null**	0	3032
**nbr.na**	0	0
**min**	1.023e-06	0
**max**	733.5	100
**range**	733.5	100
**sum**	1183168	865823
**median**	48.5	44.39
**mean**	58.6	42.89
**SE.mean**	0.3052	0.1998
**CI.mean.0.95**	0.5983	0.3917
**var**	1881	806.3
**std.dev**	43.37	28.4
**coef.var**	0.7401	0.6621

	Bottom time doing steps (%)	Descent pitch angle (degree)
**nbr.val**	20189	20177
**nbr.null**	1411	0
**nbr.na**	0	12
**min**	0	0.01534
**max**	100	84.98
**range**	100	84.97
**sum**	544118	964088
**median**	20.59	49.3
**mean**	26.95	47.78
**SE.mean**	0.1639	0.1142
**CI.mean.0.95**	0.3213	0.2238
**var**	542.6	263
**std.dev**	23.29	16.22
**coef.var**	0.8643	0.3394

	Ascent pitch angle (degree)	Pitch angle variability at bottom
**nbr.val**	20178	20116
**nbr.null**	0	0
**nbr.na**	11	73
**min**	0.09414	7.627e-05
**max**	85.7	0.5756
**range**	85.6	0.5756
**sum**	1107008	2657
**median**	56.29	0.1255
**mean**	54.86	0.1321
**SE.mean**	0.1022	0.0005017
**CI.mean.0.95**	0.2003	0.0009834
**var**	210.8	0.005063
**std.dev**	14.52	0.07116
**coef.var**	0.2646	0.5387

	Pitch angle variability at bottom	Heading angle variability at bottom
**nbr.val**	20116	20116
**nbr.null**	0	0
**nbr.na**	73	73
**min**	7.627e-05	0.0001091
**max**	0.5756	0.9955
**range**	0.5756	0.9954
**sum**	2657	8101
**median**	0.1255	0.3743
**mean**	0.1321	0.4027
**SE.mean**	0.0005017	0.001649
**CI.mean.0.95**	0.0009834	0.003233
**var**	0.005063	0.05473
**std.dev**	0.07116	0.2339
**coef.var**	0.5387	0.5809

	Dive duration (s)
**nbr.val**	20189
**nbr.null**	0
**nbr.na**	0
**min**	306
**max**	5064
**range**	4758
**sum**	22264654
**median**	1096
**mean**	1103
**SE.mean**	2.17
**CI.mean.0.95**	4.252
**var**	95025
**std.dev**	308.3
**coef.var**	0.2795

III - Prey Encounter Events (PEE) in non-drift dives

- Percentage of non-drift dives with PEE = 91.08 %.
- Percentage of PEE in descent = 10.48 %.
- Percentage of PEE in bottom = 78.03 %.
- Percentage of PEE in ascent = 11.49 %.

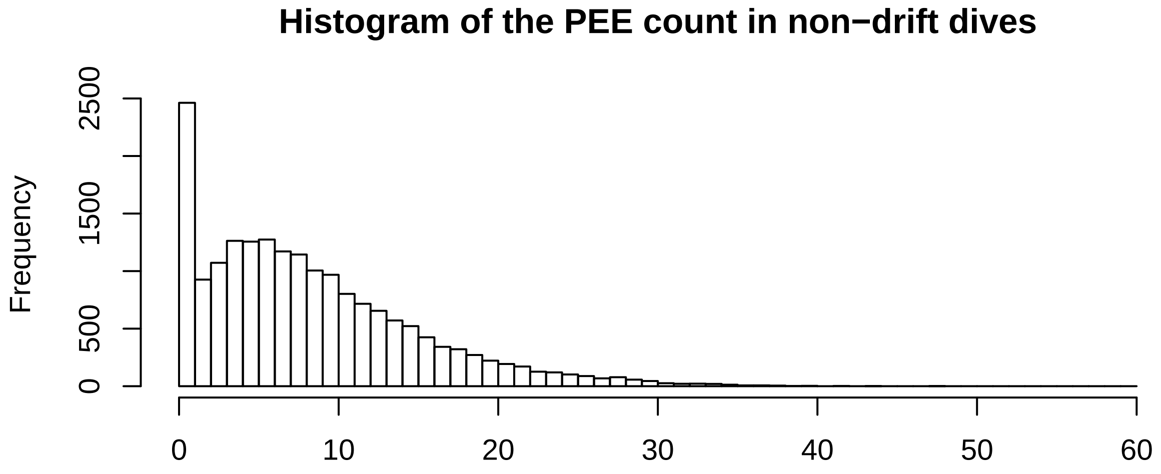

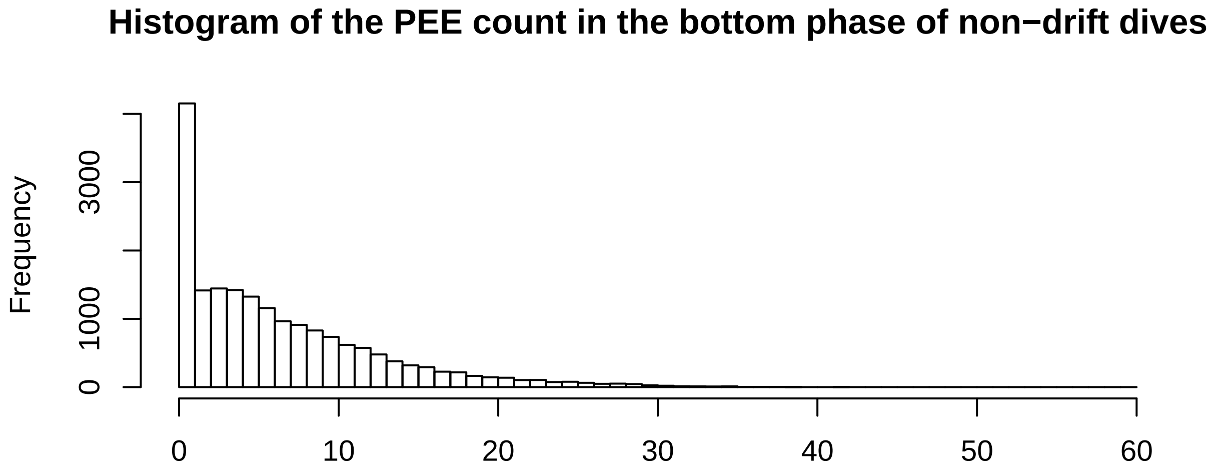

Figure A: PEE count in non drift dives: complete dives or dives' bottom phase only.
