# Supplementary material for: How Elephant Seals (Mirounga leonina) Adjust Their Fine Scale Horizontal Movement and Diving Behaviour in Relation to Prey Encounter Rate
Source: PLoS One. 2016 Dec 14;11(12):e0167226. doi: 10.1371/journal.pone.0167226 (PMC5156345; doi:10.1371/journal.pone.0167226)
Supplement: S2 Appendix — (PDF) [file pone.0167226.s002.pdf]

S2 Appendix: Detection of Prey Encounter Events (PEE)

I - Can we detect PEE using a back-mounted accelerometer?

We used back-mounted to detect PEE instead of head-mounted accelerometers (Wildlife Computers Daily Diary Tags, see device deployement details in the S1 appendix). Here we provide the evidence that this approach yields results very similar to head-mounted accelerometers.

An individual (2010-21) was equipped with two accelerometers, on its head and back, allowing comparison of the results of PEE detection method according to the logger attachment. Each accelerometer has its own clock. A small difference between the clock's internal frequencies lead to a drift between the time of the loggers. Cumulated over weeks the lag between the loggers' time is large enought to be an issue when comparing events at fine temporal scales. Synchronization (Figure A) is accomplished by finding the time lags which maximize the cross-correlation between the depth sequences of two loggers in regularly-spaced intervals along the time series.

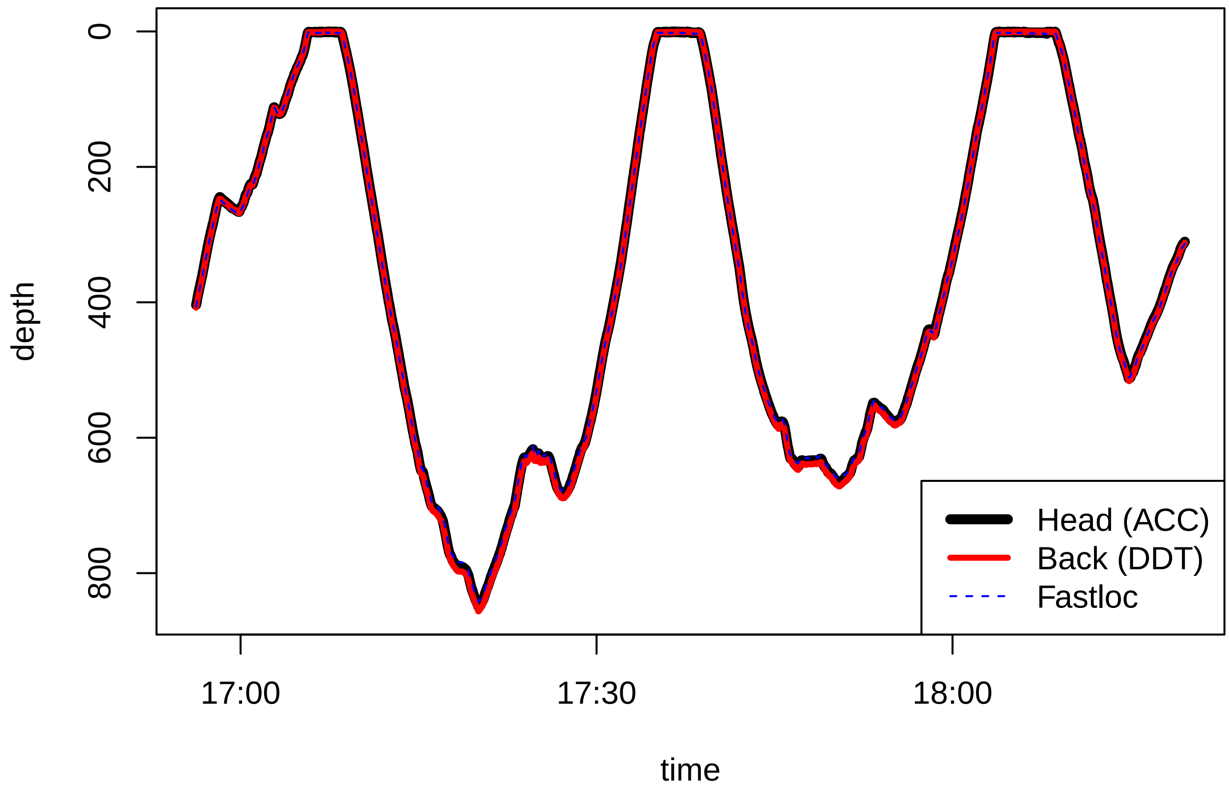

Figure A: Random sample of the datasets showing synchronization of the devices attached to the individual 2010-21: the two accelerometers (ACC and DDT) and the location collector device (Fastloc). Pearson's product moment correlation coefficient (cor.test) between the two accelerometers time-depth sequences > 0.9999.

A PEE is occuring when the 3 axes of the accelerometer are simultaneouly flagged in a “high-state” according to a 2-means clustering applyied to each axe (See methods in the article methodology, code provided at the end of this this appendix). Comparison of the 2-mean classification results obtained for the two datasets (**at 1 Hz** which requires an accurate synchronization) indicates a Pearson’s correlation coefficient of 65 %. Measures consecutively flagged as part of PEE are considered as belonging to a same PEE. Here we provide a comparison of the **count of PEE per dive** which is more robsut to time lag between datasets and is more similar to the metric used in the paper analyses. From the quantile-quantile plot it is noticeable that the PEE count according to the two accelerometers tend to differ in the range of high values (> 20) where some PEE are not detected with the back-mounted accelerometer (Figure B, left). While PEE detection is expected to work best with head-mounted accelerometers, the results obtained with back mounted accelerometer are really close. The difference mentionned in the range of high PEE counts is not really noticeable on a classic scatter plot (Figure B, right). The overall Pearson’s product moment correlation coefficient between the two PEE count data series is equal to 93%.

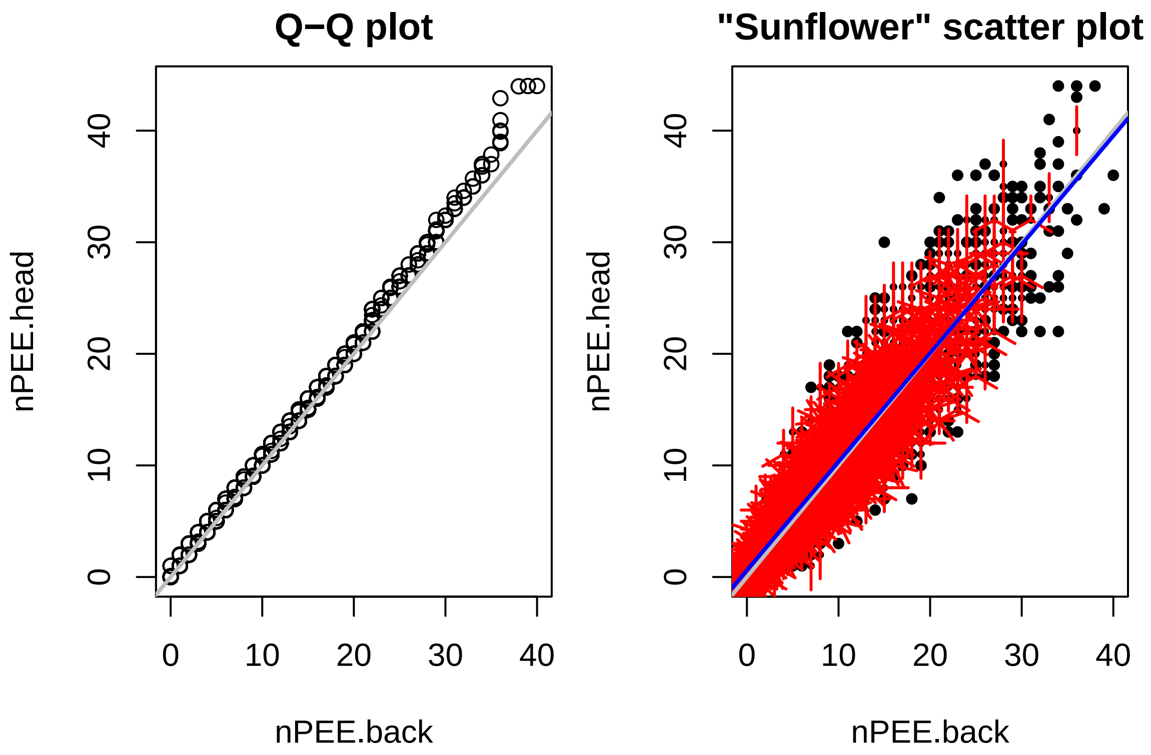

Figure B: Left: Quantile-Quantile plot of the PEE count per dive calculated on the two datasets. Right: Sunflowerplot between PEE count per dive calculated on the two datasets. Black dot indicates 1 observation. Red ticks indicates the number of superimposed observations. Grey line stands for identity (y = x). Blue line displays the best linear regression fit (see estimated parameters in Table A).

	Estimate	Std. Error	t value	Pr(>|t|)
**(Intercept)**	0.598	0.07142	8.373	7.482e-17
**nb PEE back**	0.9733	0.005713	170.4	0

Table A: Coefficients of the linear regression fit between nPPE.head and nPEE.back (blue line on Figure 2-right, r-squared = 87 %).

II - R code for Prey Encounter Events detection

```r
#' Identify Prey Catch Attempts / Prey Encounter Events
#'
#' @param x Data table with time in 1st column and 3D acceleration
#' in the next columns. Variables must be named "time", "ax", "ay", and "az"
#' @param fs sampling frequency of the input data (Hz).
#' @param fc Cut-off frequency for the butterworth high pass filter (Hz)
#' @return returns a logical vector of prey catch attempts at 1 Hz frequency.
#' @source Source code rbl package: \url{https://github.com/SESman/rbl}
prey_catch_attempts <- function(x, fs = 16, fc = 2.64) {
  stopifnot(require("data.table"))
  stopifnot(require("signal"))
  stopifnot(require("RcppRoll"))
  # Generate Butterworth filter
  # Critical frequencies of the filter: f_cutoff / (f_sampling/2)
  bf_pca  <- butter(3, W = fc / (0.5*fs), type = 'high')
  # Apply filter
  if (!is.data.table(x)) x <- data.table(x, key = "time")
  .f <- function(x) as.numeric(filtfilt(bf_pca, x))
  x <- x[ , 2:4 := lapply(.SD, .f), .SDcols = 2:4]
  gc()

  # 1 s fixed window standard deviation + aggregate data to 1 Hz
  x <- x[ , lapply(.SD, sd, na.rm = TRUE), by = time]
  # In case of NAs set ACC to zero
  nas <- lapply(x[ , 2:4, with = FALSE], is.na)
  nas_vector <- Reduce("|", nas)
  if (any(nas_vector)) {
    warning("NAs found and replaced by 0. NA proportion:", mean(nas_vector))
    x$ax[nas$ax] <- 0
    x$ay[nas$ay] <- 0
    x$az[nas$az] <- 0
  }
  gc()

  # 5 s moving window standard deviation
  .f <- function(x) c(0,0,roll_sd(x, 5),0,0)
  x <- x[ , 2:4 := lapply(.SD, .f), .SDcols = 2:4]
  gc()

  # kmean clutering: "high" = TRUE vs "low" = FALSE
  .f <- function(x) {
    km_mod <- kmeans(x, 2)
    high_state <- which.max(km_mod$centers)
    as.logical(km_mod$cluster == high_state)
  }
  x <- x[ , 2:4 := lapply(.SD, .f), .SDcols = 2:4]

  # Aggregate and return to data.frame
  # records classified as PCA if the three axis are simultaneously in high state
  Reduce("&", x[ , time := NULL])
}
```
