# Supplementary material for: How Elephant Seals (Mirounga leonina) Adjust Their Fine Scale Horizontal Movement and Diving Behaviour in Relation to Prey Encounter Rate
Source: PLoS One. 2016 Dec 14;11(12):e0167226. doi: 10.1371/journal.pone.0167226 (PMC5156345; doi:10.1371/journal.pone.0167226)
Supplement: S3 Appendix — (PDF) [file pone.0167226.s003.pdf]

S3 Appendix: Surface horizontal speed

I - Relationship with bottom prey encounter event (PEE) rate

Transit rate (or horizontal speed measured from the ARGOS/GPS track) is known to decrease with the foraging activity. For elephant seals this relashionship have been used to infer the location of Areas of Restricted Search were the animals were assumed to be foraging. We define the “surface horizontal speed” as the average horizontal speed during a dive. It is measured fom GPS location collected during the surface periods imediatly preceding and following a dive. Here we present relashionship between the “surface horizontal speed” and the PEE rate for the two individuals equipped with GPS loggers (2012-09 and 2010-21), that we used to describe the effect of diving behaviours on the horizontal speed at surface (model 2).

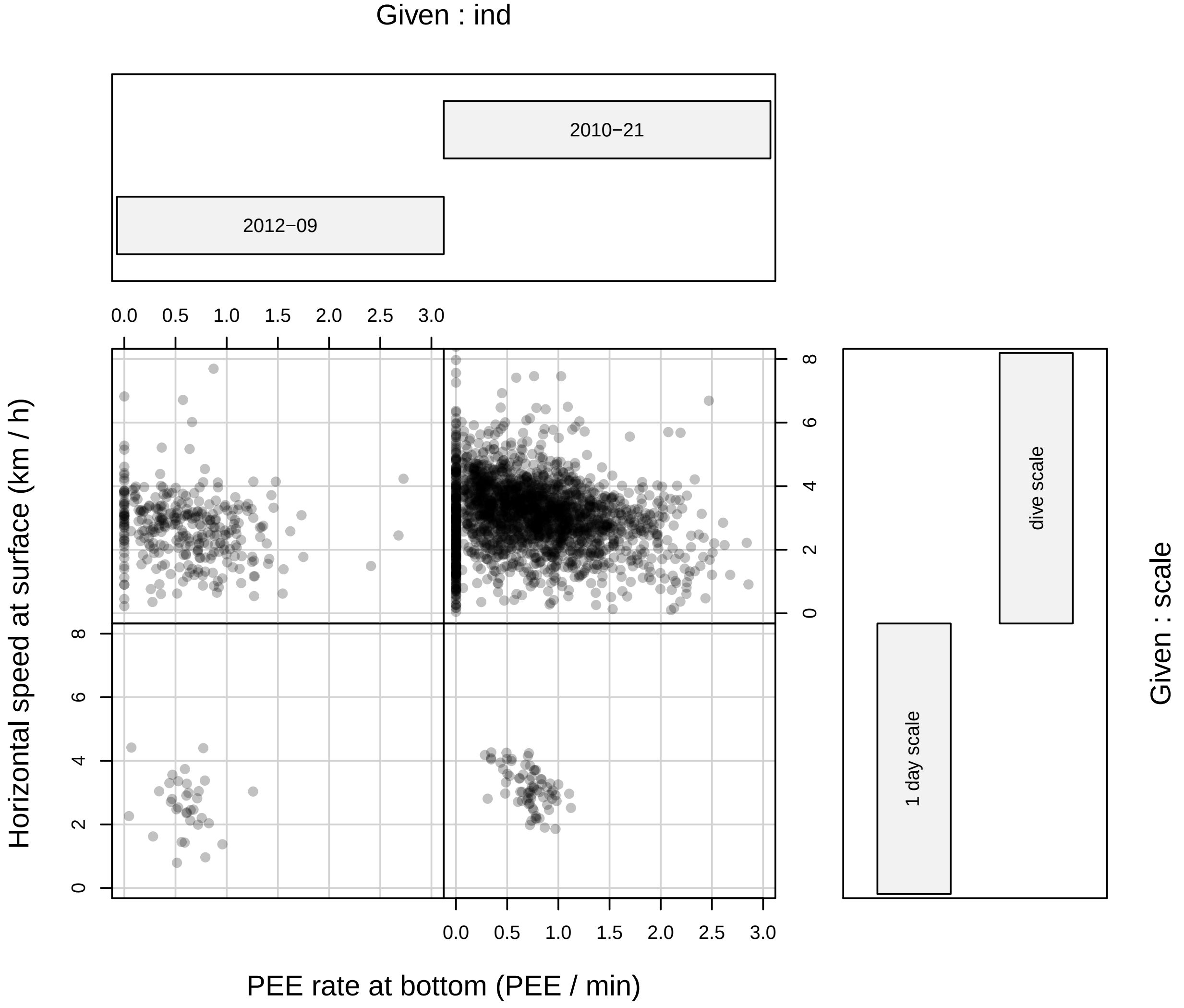

Figure A: Relashionship between the surface horizontal speed and the PEE rate at dive scale and 1 day scale Dive scale: each point was obtained from a dive surrounded by two observed GPS locations. One day scale: each point is obtained by averaging the dive scale observations every 24 hours.

II - Does the selection of GPS-located dive induce some bias toward long surface durations ?"

In order to investigate how horizontal surface speed relates to diving behavior (model 2), we selected only dives associated with GPS locations in the surface periods preceding and following the dive. Here we test if this selection could introduce some bias toward dives with longer surface periods.

The duration of surface periods with successful GPS location was found to last longer than surface periods without GPS location by 7 seconds (Table 1). We acknowledge that we did not account for this bias in our study.

```
## About surface duration and amount of GPS-located dives:
##  Percentage of dives with a location in the preceding surface:  59.60723 %
##  Percentage of dives with a location in preceding & following surface:  39.37156 %

##   Quantiles of surface duration (s):

##      0%      1%      5%     95%     99%    100%
##   21.00   84.00   93.00  162.00 1409.73 9330.00

##  Average surface duration:  158.6743 s
##  Standard deviation of surface duration:  382.7328 s
```

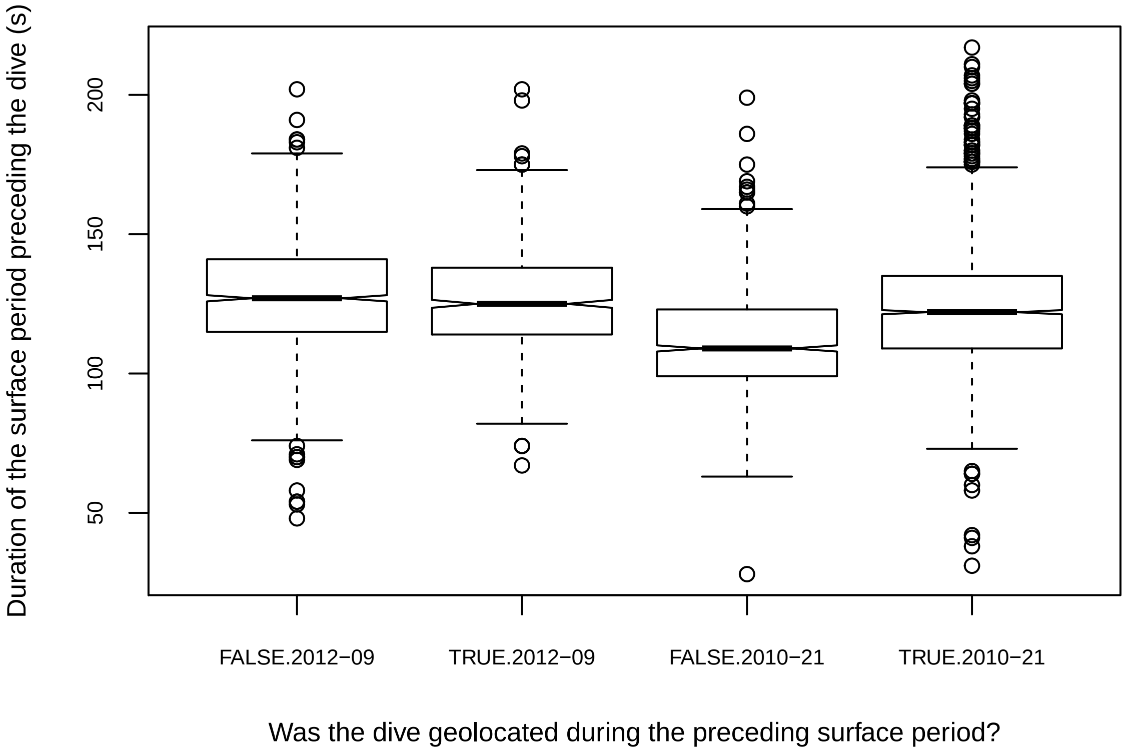

Figure B: Comparison of surface period duration in relation with the collection of a GPS location (for the two individual equipped with GPS). 2012-09 and 2010-21 are the identifier of the two individuals equipped with GPS loggers.

Table A: Fixed effects: psf.dur ~ is_geoloc

	Value	Std.Error	DF	t-value	p-value
**(Intercept)**	119.8	5.186	6213	23.1	2.339e-113
**is_geolocTRUE**	6.837	0.5143	6213	13.29	8.687e-40

Table B: Standardized Within-Group Residuals

Min	Q1	Med	Q3	Max
-4.855	-0.6853	-0.07492	0.6372	4.859

Table C: Summary of the mixed model Surface duration = f(GPS-location, individual) where individual is a random intercept effect.

	Observations	Groups	Log-restricted-likelihood
**ind**	6216	2	-27170

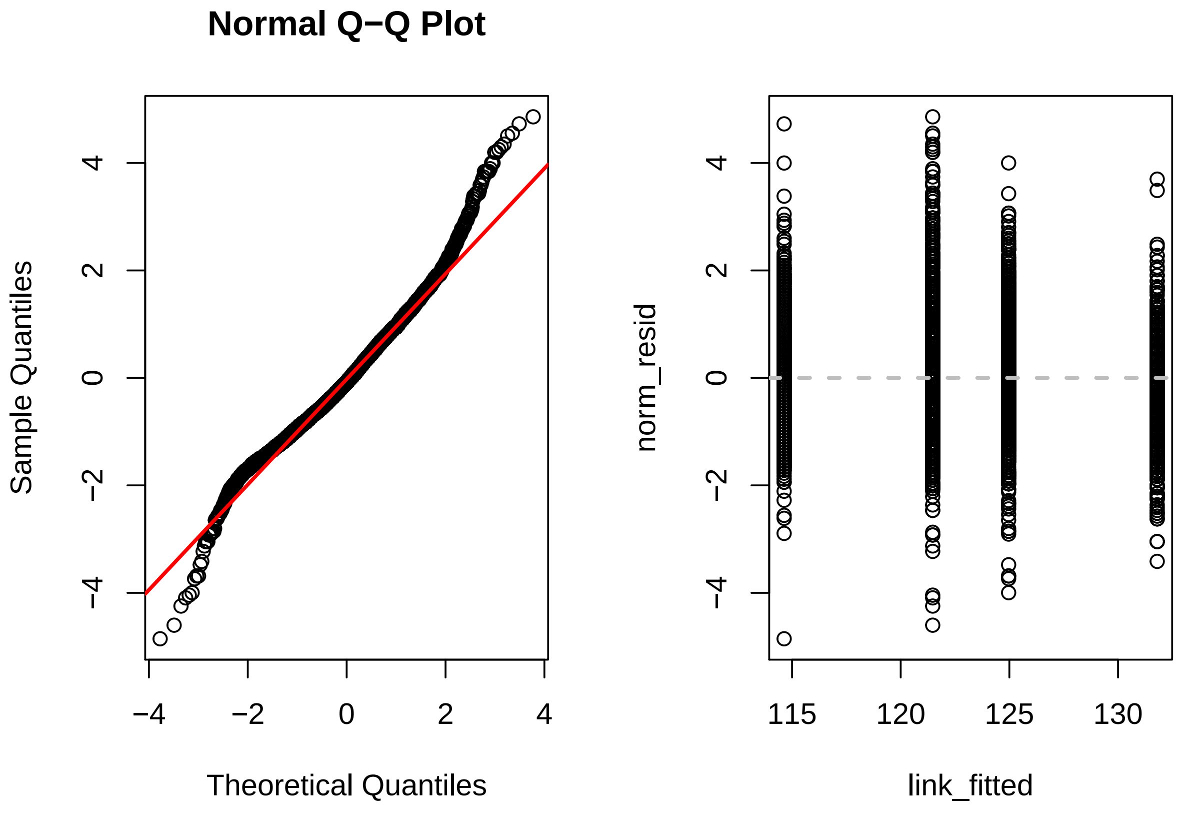

Figure C: Model residuals
