# Supplementary material for: How Elephant Seals (Mirounga leonina) Adjust Their Fine Scale Horizontal Movement and Diving Behaviour in Relation to Prey Encounter Rate
Source: PLoS One. 2016 Dec 14;11(12):e0167226. doi: 10.1371/journal.pone.0167226 (PMC5156345; doi:10.1371/journal.pone.0167226)
Supplement: S4 Appendix — (PDF) [file pone.0167226.s004.pdf]

S4 Appendix: Influence of vertical speed on swimming effort during transit phases

This appendix is a complement to the main paper analysis related to transit speed and diving efficiency adjustments (models 3 & 4). Our goal is to detail how the vertical speed adjustements made by SES during their transit phases impact on the swimming effort required to acomplish these transits.

I - Data summary per phase

Table A: Data summary for descent phases.

dur	max_depth	tot_swm_eff	vert_spd
Min. : 41.0	Min. : 48.05	Min. : 3.182	Min. :0.1745
1st Qu.:177.0	1st Qu.: 259.50	1st Qu.: 20.096	1st Qu.:1.2199
Median :271.0	Median : 373.13	Median : 32.592	Median :1.5391
Mean :289.7	Mean : 398.09	Mean : 45.754	Mean :1.5021
3rd Qu.:379.0	3rd Qu.: 532.13	3rd Qu.: 54.272	3rd Qu.:1.8176
Max. :987.0	Max. :1051.43	Max. :372.188	Max. :2.7342

Table B: Data summary for ascent phases.

dur	max_depth	tot_swm_eff	vert_spd
Min. : 22.0	Min. : 56.55	Min. : 36.01	Min. :0.1565
1st Qu.:203.0	1st Qu.: 271.23	1st Qu.: 255.97	1st Qu.:1.2186
Median :299.0	Median : 387.46	Median : 390.54	Median :1.3954
Mean :315.4	Mean : 411.42	Mean : 402.94	Mean :1.3640
3rd Qu.:417.0	3rd Qu.: 546.60	3rd Qu.: 532.87	3rd Qu.:1.5545
Max. :970.0	Max. :1051.43	Max. :1031.96	Max. :2.9047

II - Exploratory plots

1. Swimming effort is higher in ascent than in descent (Figure A & B).
2. Effect of transit duration is stronger for ascent phases (Figure A). This seems logical because, according to the previous point, the ascent phase requires a greater swimming effort. Yet, considering a multiplicative effect of duration (Figure B where trends according to duration are very similar for ascent and descent), the transit duration has roughly the same effect in ascent and descent phases.
3. The effect of vertical speed seems to accentuate for longer transit time (Figure A). Again, this is logical because the swimming effort variations due to changes in the vertical speed are magnified over the complete transit duration. As in the previous point, this trend is not noticeable when considering a multiplicative effect (for a given phase, on Figure B, the slopes according to vertical speed are similar across the different durations).
4. For descents, a “>” shape is noticeable (Figure B) and seems to indicate that an increase in descent vertical speed could be either performed passively (e.g. by pitch angle adjustments) or actively (e.g. by intensifying swimmming effort). However, in the following analyses we will focus on the global effect of the changes of vertical speed on the swimming effort without distinguishing the different possible mecanisms controling the transit rate.

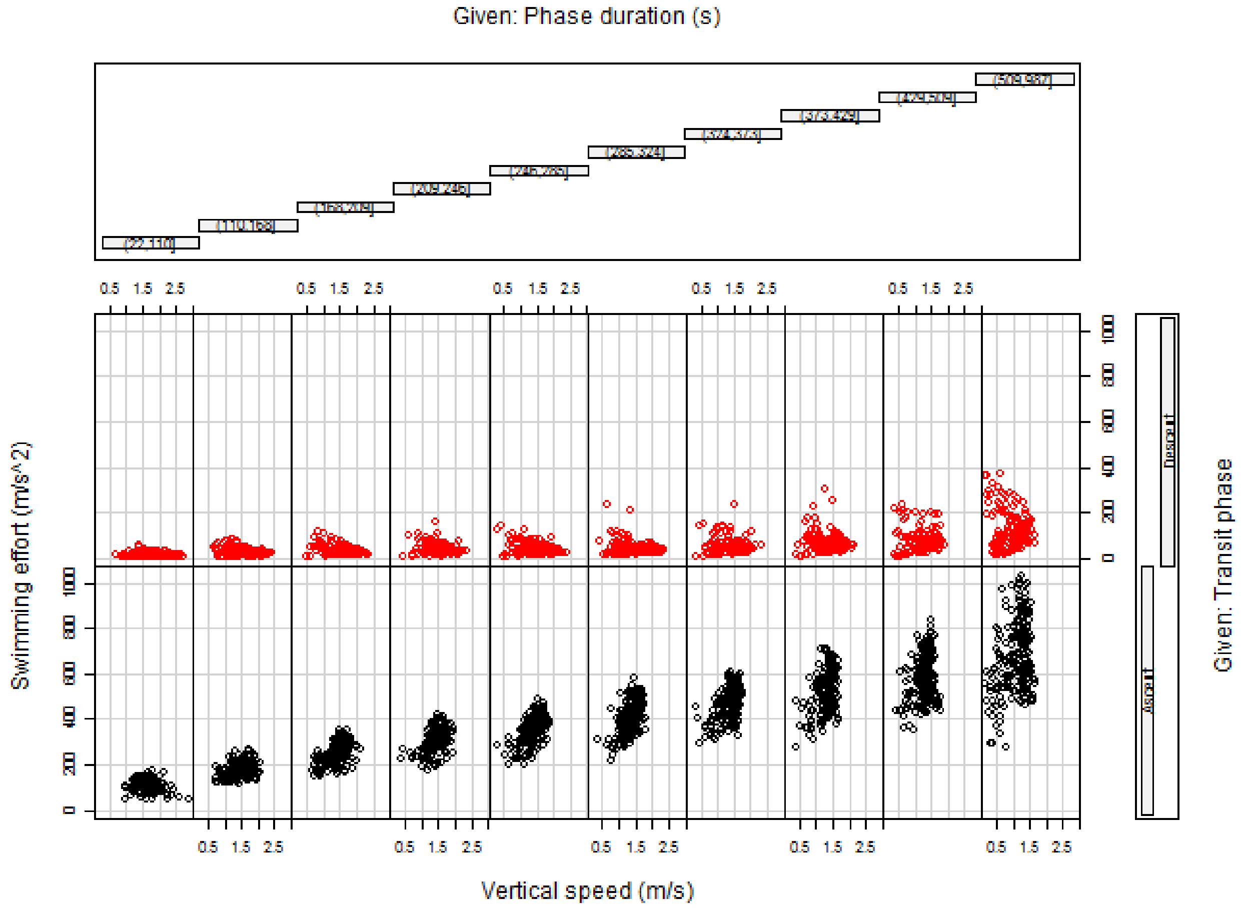

Figure A: Relationship between swimming effort, vertical spped and phase duration in the descent and ascent phases of SES dives. Ascent phase observation in black, Descent phase observations in red.

```
##
## Missing rows: 1666
```

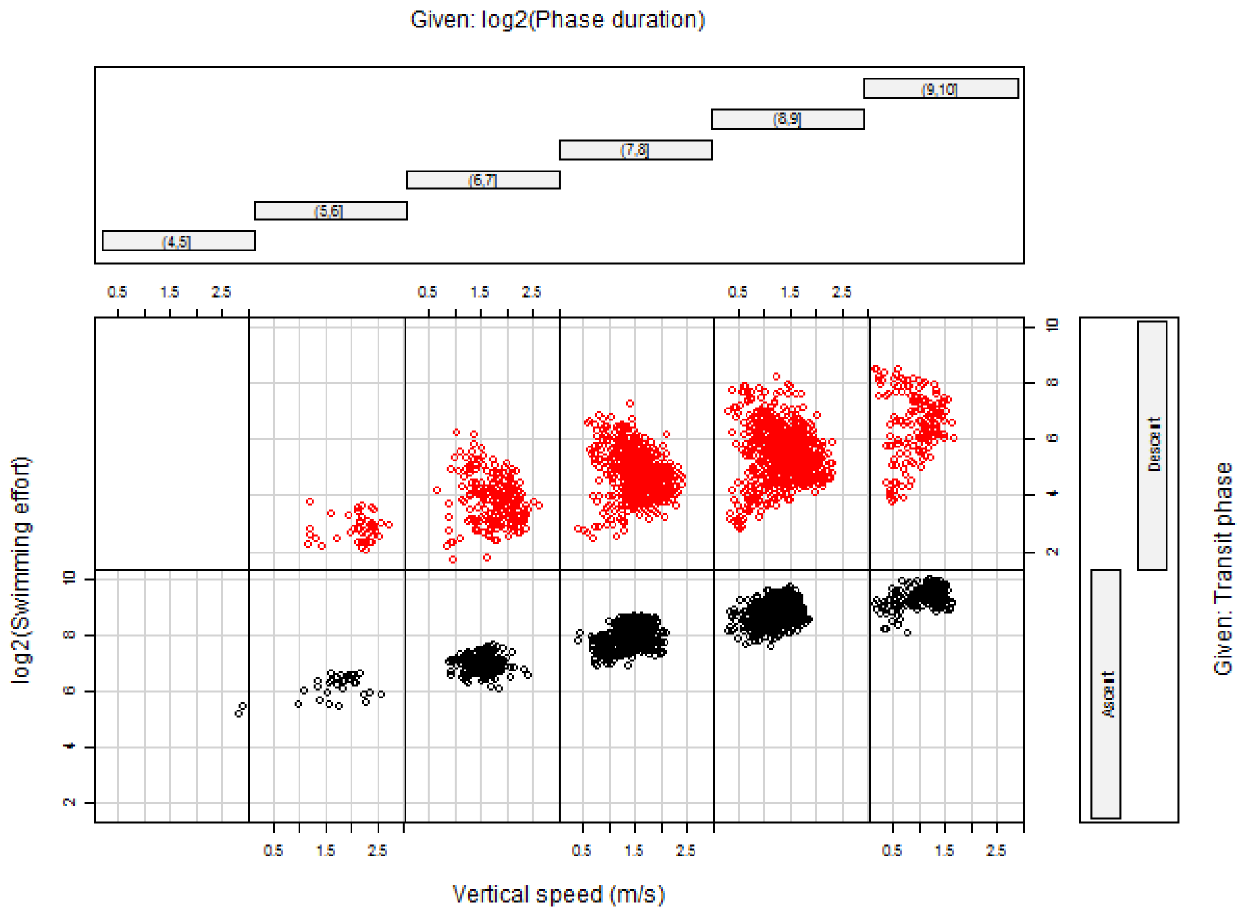

Figure B: Relationship between swimming effort (log scale), vertical spped and phase duration (log scale) in the descent and ascent phases of SES dives. Ascent phase observation in black, Descent phase observations in red.

III - Statistical analysis

Theorical considerations (and the previous comparison between Figure A & B) would recommend to model log-transformed swimming effort as a function of log-transformed duration. However, because it allows us to model the effect of depth using a simple interaction term and because the first exploratory plot (Figure A) provides the evidence that a linear (additive) effect of duration on the swimming effort yield in a reasonale fit, we opt for a model with simple linear additive effects. So far the data presented represented only one dive every ten. The model selection is based on the same data.

In a first step we selected the explanary variables to be used in the model. The variance inflation factor provided a simple criterion.

Table C: Variance Inflation Factor (VIF) table of the potential explanatory variables. Maximum depth was removed from potential explanatory variables. No information is lost because one variable is function of the others (e.g. Vertical speed = maximum depth / duration).

Variables	VIF
dur	9.061
max_depth	7.013
vert_spd	3.741

Table D: This is the final VIF table. All VIF < 5.

Variables	VIF
dur	1.301
vert_spd	1.301

Then we used a stepwise AIC model selection procedure. Starting from the null model the algorithm was allowed adding or droping a variable at any step untils it reaches the null model (all variables dropped), the full model (effect of the phase factor, of the phase duration and of the vertical speed as well as of the interaction between duration and vertical speed) or it minimizes the AIC. In R syntax the full model specification was `tot_swm_eff ~ phase + (phase:dur) * (phase:vert_spd)`. Each time the stepwise algorithm reached a minimum, the correponding model was re-adjusted with the REML algorithm which is supposed to provide better parameter estimates.

However, the model we obtained after this step had serious heteroskedasticity issues as well as a residual normality issue. Thus, we tested various correlation structure to handle the heteroskedasticity of our data. The package “nlme” provides many tools to help with this task. Among the various family tested (“varIdent”, “varFixed”, “varPower” and “varExp” and “varConstPower”) we had the higher success with the “varConstPower” family where the “Power” was able to handle the continuous increase of variance with the predicted values and the “Constant” part the estimation of different parameter for ascent and descent phases. This step lowered the AIC by 100 points. The stepwise algorithm was implemented again using this correlation structure.

Once the variance structure was selected we tested if random effects could improve our model (lowering the AIC). Among the combinations of random slopes and intercepts that we tried to fit, the most complex (in terms of number of parameters) failed to converge (even when increasing by four times the number of maximum iterations allowed to likelihood maximization). The smallest AIC was obtained when setting dive phase as random slopes and intercepts. The stepwise AIC selection was implemented again using the correlation structure and these random effects yielding the final model that we will now present (Table E-G).

Table E: Fixed effects: tot_swm_eff ~ phase + phase:dur + phase:vert_spd + phase:dur:vert_spd

	Value	Std.Error	DF	t-value	p-value
**(Intercept)**	-21.41	10.1	3826	-2.12	0.03407
**phaseDescent**	33.37	10.12	3826	3.298	0.0009823
**phaseAscent:dur**	0.7676	0.02169	3826	35.38	1.659e-237
**phaseDescent:dur**	0.1589	0.01397	3826	11.37	1.699e-29
**phaseAscent:vert_spd**	9.228	3.045	3826	3.03	0.002461
**phaseDescent:vert_spd**	-6.259	1.523	3826	-4.109	4.05e-05
**phaseAscent:dur:vert_spd**	0.4151	0.0157	3826	26.44	1.281e-141
**phaseDescent:dur:vert_spd**	-0.01203	0.008303	3826	-1.449	0.1473

Table F: Standardized Within-Group Residuals

Min	Q1	Med	Q3	Max
-4.233	-0.614	-0.06028	0.5712	7.189

Table G: Linear mixed-effects model fit by REML : tot_swm_eff ~ phase + phase:dur + phase:vert_spd + phase:dur:vert_spd

	Observations	Groups	Log-restricted-likelihood
**ind**	3842	9	-18571

The coefficients estimates and t-statistics for the last model fitted presented in Table E. The squared Pearson correlation coefficient between predicted and observed response is equal to 0.9530408.

IV - Result & discussion

Discussion about the effects of covariate directly from this summary table is difficult because of the numerous interaction terms. For the sake of clarity, we will present and discuss the result using graphics (Figure C).

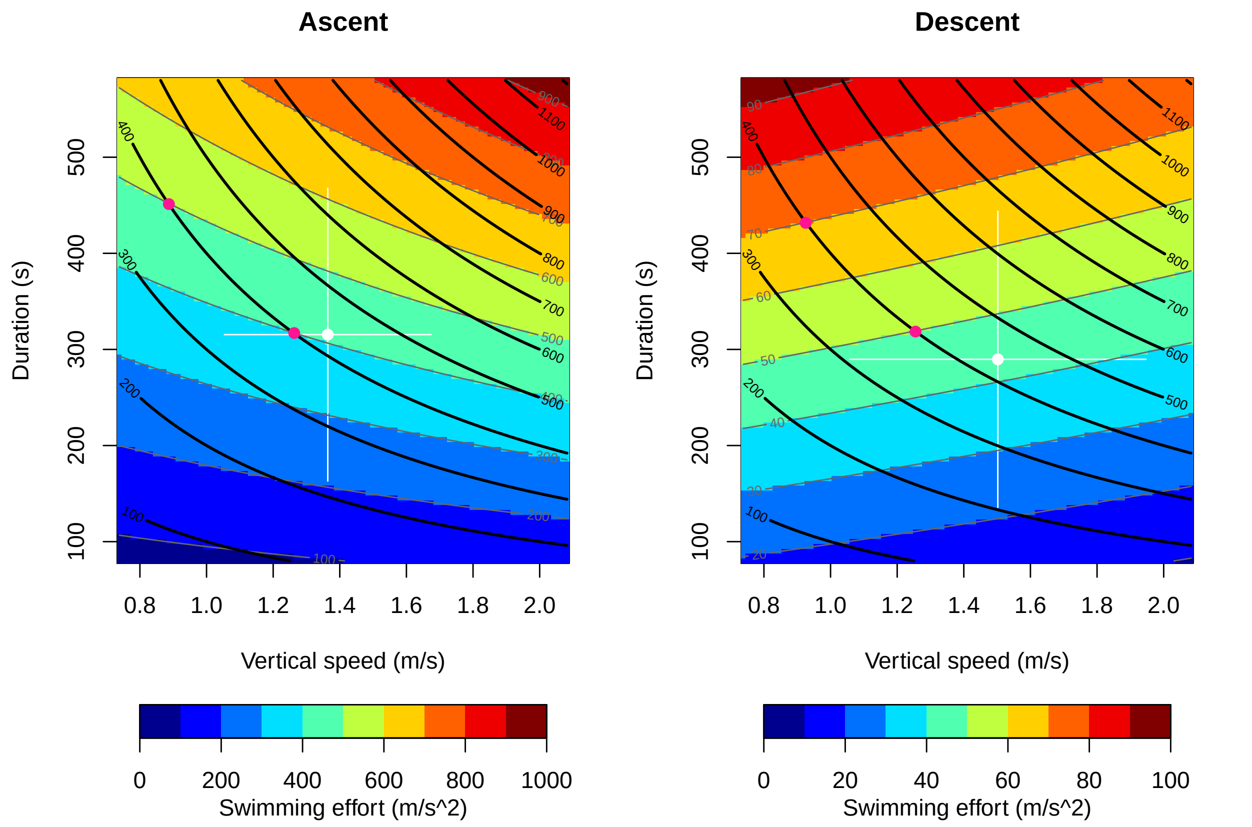

Figure C: Image plots of model preditions for ascent and descent phases. The thin grey lines are "iso swimming effort" contour lines. The thick black lines are "iso depth" contour lines. To help with the location of likely occuring values of the covariates, the white dot indicates the average vertical speed and phase duration in our observations dataset. White segments display the +- 1 SD range around these means.

In both ascents and descents the duration is the main determinant of the cumulated swimming effort deployed by the seals to perform their transit. The effect of the vertical speed on the swimming effort qualitatively differs according to ascent or descent. Indeed, the ascent phase has greater swimming effort requirements than the descent phase. For a given duration, increasing the vertical speed implies a greater swimming effort in ascent but tend to be associated with a smaller swimming effort in descent (yet, this coefficient is not significant, see "phaseDescent:dur:vert_spd" in the previous model summary table). This leads to greater overall swimming efforts in ascent compared to descents (as highlighted on the figure by the color scales and white dots). This can be explained by the fact that all our individuals are post-breeding female SES, hence negatively buoyant, which can speed up their descent rate by increasing their diving angle while gliding down to the bottom of their dive.

The duration and the vertical speed are physically related. As SES increase their speed to transit from/to surface to/from the bottom of a dive, the duration of their transit is diminuished. More pragmatically, we can look at the changes of swimming effort according to transit speed *given a target depth* by following the path of "iso-depth" lines. For example, considering a SES diving at 400 m to perform the bottom phase:

- **In ascent:** According to the model, a vertical speed of 0.89 m/s would imply a swimming effort of 500 m/s^2 (pink point on the left) and a vertical speed of 1.26 m/s would imply a swimming effort of 400 m/s^2 (pink point on the right). This increase of the vertical speed by 42 % translates into a decrease of the total swimming effort of 20 %.
- **In descent:** This principle applies again with more strength. A vertical speed of 0.93 m/s would require a swimming effort of 70 m/s^2 and vertical speed of 1.26 m/s would require a swimming effort of

50 m/s^2. This increase of the vertical speed by 36 % translates into a decrease of the total swimming effort of 29 %.

- The larger is the gradient of swimming effort along the “iso-depth” path, the greater are the savings of swimming effort with increasing vertical speed. According to this model, the beneficial compensation effect of a reduced duration with increasing speed is attenuated toward fast vertical speeds and short phase durations, particularly during ascent phases.

To sumarize: When negatively buoyant SES speed up their ascent rate, they increase their *swimming effort per unit of time* but the overall savings of transit duration compensate for it and transforms in a reduced or similar swimming effort cumulated over the whole period. In descent, the same compensation phenomenon occurs with more strength since the cost of an increased vertical speed in terms of swimming effort per unit of time is lower than in ascent.

V - Model diagnostic plot

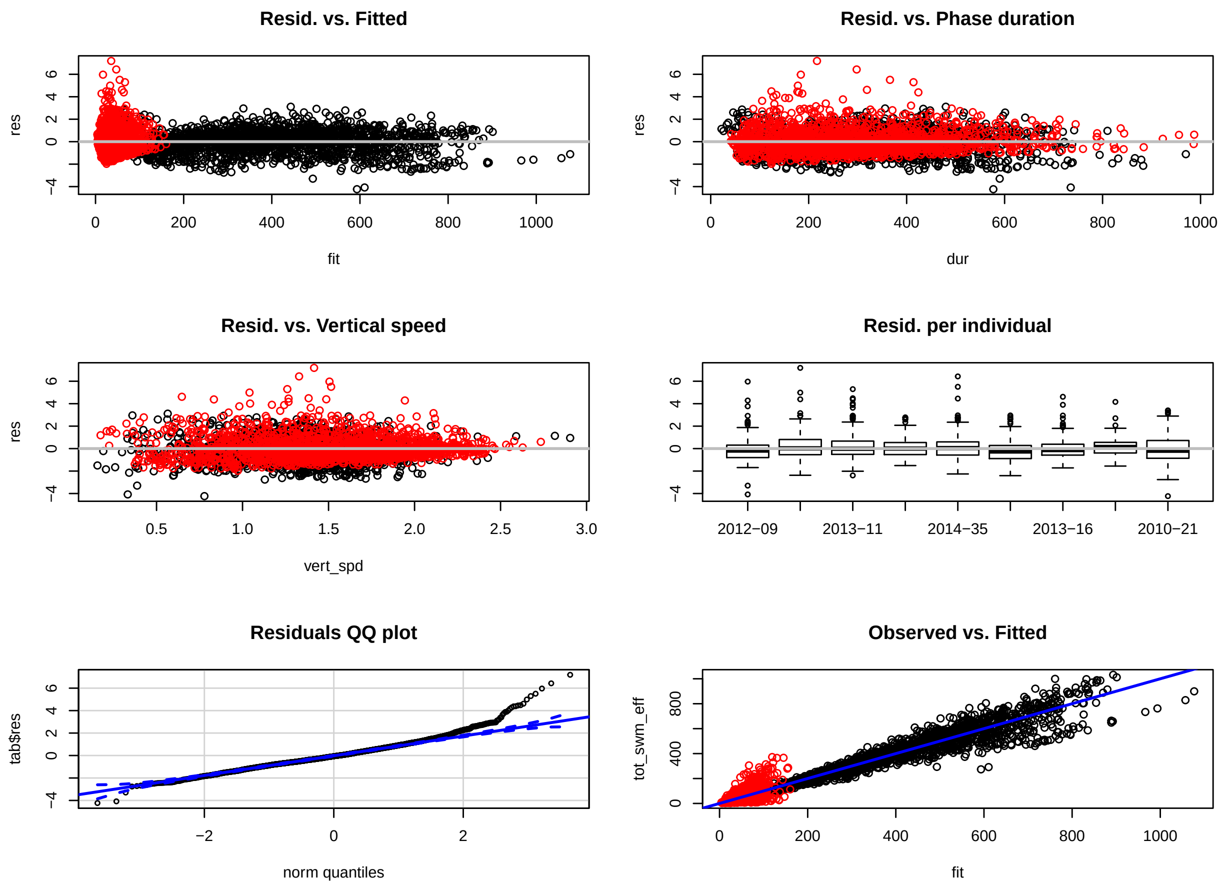

Figure D: Diagnostic plot of the final model
